# A hybrid transcriptome assembly reveals coding and noncoding landscapes in *Drosophila* hemocytes

**DOI:** 10.1093/genetics/iyag208

**Published:** 2026-08-11

**Authors:** Kyung-Tae Lee, Bumsik Cho, Daewon Lee, Sunggyu Yoon, Hobin Do, Jaeseong Han, Hansoll Na, Sangho Yoon, Mingyu Shin, Jiwon Shim, Jin-Wu Nam

**Affiliations:** Department of Life Science, College of Natural Sciences, Hanyang University, Seoul 04763, Republic of Korea; Bio-BigData Center, Hanyang Institute of Bioscience and Biotechnology, Hanyang University, Seoul 04763, Republic of Korea; Hanyang Institute of Advanced BioConvergence, Hanyang University, Seoul 04763, Republic of Korea; Division of Oncology, Department of Medicine, School of Medicine, Stanford University, Stanford, CA 94305, United States; Department of Life Science, College of Natural Sciences, Hanyang University, Seoul 04763, Republic of Korea; Department of Life Science, College of Natural Sciences, Hanyang University, Seoul 04763, Republic of Korea; School of Biological Sciences, Seoul National University, Seoul 08826, Republic of Korea; Department of Life Science, College of Natural Sciences, Hanyang University, Seoul 04763, Republic of Korea; Department of Life Science, College of Natural Sciences, Hanyang University, Seoul 04763, Republic of Korea; Department of Life Science, College of Natural Sciences, Hanyang University, Seoul 04763, Republic of Korea; Department of Life Science, College of Natural Sciences, Hanyang University, Seoul 04763, Republic of Korea; Department of Life Science, College of Natural Sciences, Hanyang University, Seoul 04763, Republic of Korea; Bio-BigData Center, Hanyang Institute of Bioscience and Biotechnology, Hanyang University, Seoul 04763, Republic of Korea; Hanyang Institute of Advanced BioConvergence, Hanyang University, Seoul 04763, Republic of Korea; Department of Life Science, College of Natural Sciences, Hanyang University, Seoul 04763, Republic of Korea; School of Biological Sciences, Seoul National University, Seoul 08826, Republic of Korea; Department of Life Science, College of Natural Sciences, Hanyang University, Seoul 04763, Republic of Korea; Bio-BigData Center, Hanyang Institute of Bioscience and Biotechnology, Hanyang University, Seoul 04763, Republic of Korea; Hanyang Institute of Advanced BioConvergence, Hanyang University, Seoul 04763, Republic of Korea; Research Institute for Convergence of Basic Sciences, Hanyang University, Seoul 04763, Republic of Korea

**Keywords:** *Drosophila*, hemocytes, single-cell transcriptome, noncoding RNA, long-read sequencing, lncRNA, lamellocyte, alternative polyadenylation, parasitic wasp, innate immunity

## Abstract

Innate immune responses triggered by *Drosophila* larval hemocytes have been extensively characterized. However, the full extent of transcriptional and posttranscriptional regulation underlying these processes remains poorly understood. Here, we employed a hybrid sequencing strategy integrating Oxford Nanopore long-read and Illumina short-read sequencing to provide a more comprehensive transcriptome annotation. This enabled the discovery of full-length transcripts with novel 5′ and 3′ boundaries and uncovered 349 previously unannotated long noncoding RNAs highly induced during late stages of wasp infestation. To ensure high-confidence transcript models, we further eliminated potential intra-priming artifacts specific to long-read cDNA data. This highly confident full-length transcript models helped reveal cell type–specific long noncoding RNA (lncRNA) markers in lamellocytes and crystal cells by single-cell analyses, which recapitulated hemocyte differentiation trajectories. Notably, RNAi-based depletion of 2 highly induced lncRNAs impaired lamellocyte formation under wasp infestation, highlighting their functional relevance. Collectively, our findings provide detailed insights into the *Drosophila* larval immune transcriptomes through the long-read sequencing and highlight the regulatory roles of noncoding RNAs in innate immunity.

## Teaser

Drosophila mount powerful immune responses against parasites, but the genetic programs controlling these responses remain incompletely understood. Lee et al. used a cutting-edge DNA sequencing approach combining two complementary technologies to build a comprehensive map of gene activity in Drosophila immune cells during parasitic wasp attack. This revealed 349 previously unknown long non-coding RNAs that are strongly activated during infection. The authors show that two of these non-coding RNAs are essential for producing the specialized immune cells that neutralize wasp parasites. These findings reveal a hidden layer of gene regulation controlling innate immunity, with broad implications for understanding immune responses across species.

## Introduction

*Drosophila melanogaster* has been widely utilized as a model organism for investigating innate immunity and myeloid cell development due to its relatively simple yet functionally robust blood cell (hemocyte) system (Gold and Brückner 2014; Banerjee et al. 2019; Morin-Poulard et al. 2021). Three major types of hemocytes—plasmatocytes (PMs), crystal cells (CCs), and lamellocytes (LMs)—cooperate to defend the host against infection and various stresses. PMs and CCs are present under steady-state conditions, whereas LMs are induced by immune activation (Rizki and Rizki 1984; Sorrentino et al. 2004; Bidla et al. 2007; Kurucz et al. 2007; Binggeli et al. 2014; Melcarne et al. 2019; Shin et al. 2024). *Drosophila* hematopoiesis occurs in 2 distinct waves—embryonic and larval. The embryonic wave generates circulating hemocytes while the larval wave produces lymph gland–derived progenitors. These cells collectively participate in immune responses throughout larval development and into adult stages (Evans et al. 2003; Sorrentino et al. 2004; Ratheesh et al. 2015; Cattenoz et al. 2021; Girard et al. 2021).

Recent technical advances, particularly single-cell RNA sequencing (scRNA-seq) (Lee et al. 2024), have advanced our understanding of *Drosophila* hematopoiesis by revealing heterogeneous subpopulations previously defined by morphology or marker gene expression. In addition, these studies have elucidated the lineage specification of the 3 major hemocytes based on transcriptome profiles, providing valuable datasets for future studies (Cattenoz et al. 2020; Cho et al. 2020; Tattikota et al. 2020; Coates et al. 2021; Girard et al. 2021; Ewen-Campen et al. 2023). These studies not only confirmed the classical hemocyte types but also revealed new subclusters with unique transcriptomic signatures and functions.

Under immune challenge by parasitic wasps such as *Leptopilina boulardi*, LMs differentiate to encapsulate and neutralize the invading wasp eggs (Rizki and Rizki 1984; Sorrentino et al. 2004; Anderl et al. 2016; Leitao et al. 2020). This rapid expansion and transformation of hemocytes is accompanied by extensive molecular changes, including large-scale transcriptional reprogramming of both protein-coding genes and noncoding RNAs (ncRNAs), as revealed by recent scRNA-seq-based catalogs (Cho et al. 2020; Tattikota et al. 2020). Interestingly, several long ncRNAs (lncRNAs) were detected at elevated levels in differentiating LMs and emerged as candidate regulators of LM differentiation,especially during wasp infestation (wIF), suggesting at a broader regulatory axis that may modulate hemocyte proliferation and cell fate determination (Kopp and Mendell 2018; Tattikota et al. 2020). However, the extent to which lncRNAs reprogram the regulatory network remains unclear, in part due to the lack of a comprehensive annotation for lncRNAs induced during an immune challenge.

Accurate transcript annotation is critical to capturing the full complexity of hemocyte gene regulation, especially in single-cell platforms that rely on 3′ tags for gene quantification (Macosko et al. 2015; Zheng et al. 2017). Incomplete or incorrect transcript boundaries result in the loss of important single-cell read information, reducing the sensitivity for gene detection and biasing downstream analyses (Pool et al. 2023). This is particularly problematic given the prevalence of tissue-specific alternative polyadenylation (APA) and isoform usage reported in *Drosophila*, which influences mRNA stability, localization, and translational efficiency (Smibert et al. 2012; Tian and Manley 2017; Chen et al. 2020). Therefore, it is essential to comprehensively identify unannotated transcript boundaries and expand the catalog of ncRNAs in *Drosophila* hemocytes, especially under immunologically stressed conditions such as wIF.

In this study, we address these gaps by employing a hybrid transcriptome assembly strategy that integrates long-read cDNA sequencing from Oxford Nanopore Technologies (ONT) and short-read RNA-seq from Illumina. Long-read cDNA sequencing with PCR amplification provided sufficient sequencing depth and coverages across RNA molecules to uncover unannotated untranslated regions (UTRs), diverse full-length splice isoforms, and novel lncRNAs involved in both steady-state and immune-activated larval hematopoiesis. Unexpectedly, we identified previously uncharacterized noncoding isoforms within protein-coding genes (Nam et al. 2016). Furthermore, we characterized the cell type–specific expression profiles of selected lncRNAs and identified their regulatory functions in LM differentiation and immune responses. Overall, these results provide an expanded *Drosophila* gene annotation and reveal new layers of noncoding RNA–mediated regulation in larval hemocytes.

## Materials and methods

### Sample preparation and Illumina RNA-seq

At 96 or 120 h after egg laying (AEL), with or without wIF, 100 to 150 *Drosophila OreR* larvae were dissected. Larvae were vortexed with glass beads (Sigma; G9268) for 1 min to collect the entire larval circulating hemocytes. Ten larvae were dissected together in 20 μL of Schneider's medium (Gibco; 21720024) and transferred to 100 μL of cold Schneider's medium. Hemocyte samples were centrifuged at 7,000 rpm and 4 °C for 5 min. After removing supernatant, 1,000 μL of Trizol (Molecular Research Center, MRC; TR 118) was added for RNA extraction. More than 1 μg of RNA was prepared for each experiment. Library preparation and sequencing were performed by Macrogen (Macrogen, Inc., Seoul, South Korea). Illumina short-read RNA-seq of normal lymph gland at 96 and 120 h AEL was obtained from a previous study (Cho et al. 2020) under the GEO accession GSE141274. RNA integrity number scores for RNA extracted and used for short-read RNA-seq are summarized in Supplementary Table 1.

### Library preparation for ONT sequencing

#### Library preparation

RNA extraction was performed as above for the Illumina RNA-seq at least twice and sequenced 3 times in a multiplexed manner. For the ONT experiment, the lymph gland at 120 h AEL was processed using the Oxford Nanopore SQK-PCS109 kit (PCS_9085_v109_revB_04Feb2019). The remaining samples (lymph gland at 96 h AEL with or without wIF, circulating blood at 96 and 120 h AEL with or without wIF) were processed using the Oxford Nanopore SQK-PCB109 kit (PCB_9092_v109_revA_10Oct2019). This kit shares the same reagent composition as the SQK-PCS109 kit but includes an additional barcoding step to enable sample multiplexing. All procedures were performed according to the manufacturer's instructions. The 120 h AEL lymph gland sample was sequenced once; the remaining samples were barcoded, pooled in equal concentration, and sequenced 3 times on MinION flow cells without washing. Due to higher fragmentation rates and overall lower base quality, sequencing reads from 96 and 120 h AEL blood samples were excluded from the first run. cDNA concentration, A260/A280, and A260/A230 ratios calculated by Nanodrop are summarized in Supplementary Table 1. Full protocols for SQK-PCS109 and SQK-PCB109 are available on the ONT website.

In brief, total RNA from each sample was added to a strand-switching buffer containing 4 μL 5× RT Buffer (ThermoFisher; EP0751), 1 μL RNaseOUT (Life Technologies; 10777019), 1 μL nuclease-free water, and 2 μL strand-switching primer (SSP, 10 μM). Next, 1 μL of Maxima H Minus Reverse Transcriptase (ThermoFisher; EP0751) was added, and the reverse transcription plus strand-switching reaction was carried out for 90 min at 42 °C, followed by a 5-min heat inactivation at 85 °C. After strand switching, PCR was performed for each sample with 5 μL of cDNA, 1.5 μL barcode primers, 18.5 μL nuclease-free water, and 25 μL 2× LongAmp Taq Master Mix (New England Biolabs, NEB; M0287). Twelve PCR cycles were run with a 10-min extension at 65 °C. Next, 1 μL of NEB Exonuclease 1 (20 units; NEB; M0293) was added to each PCR product and incubated 15 min at 37 °C, followed by 15 min at 80 °C. Samples were purified using 0.8× equivalents of resuspended AMPure XP beads (Agencourt; A63881), pooled at equal concentrations, and sequenced on the MinION Mk1B device. Sample concentration and integrity were evaluated using a NanoDrop (ThermoFisher; ND-ONE).

#### Sequencing and base calling of ONT data

Library sequencing was carried out on a laptop equipped with an Intel Core i7-8565 U CPU @ 1.80 GHz (1.99 GHz), 16GB RAM, and 1TB SSD. Fast5 files were generated by MinKNOW software and merged for base calling on a local server using Guppy (version 3.6.1-1) in high accuracy mode (GPU-accelerated, NVIDA TITAN XP).

### Hybrid transcriptome assembly

#### Illumina RNA-seq data processing

Illumina RNA-seq reads were initially assessed for quality using FastQC (version 0.11.8; http://www.bioinformatics.babraham.ac.uk/projects/fastqc), then trimmed by Sickle (version 1.33; https://github.com/najoshi/sickle) based on Phred-scaled quality scores. Filtered reads were aligned to the *D. melanogaster* reference genome, Berkeley Drosophila Genome Project (BDGP) release 6^2015^ using STAR (version 2.5.3a) (Dobin et al. 2013). *De novo* transcriptome assembly was performed via StringTie (version 2.1.3) (Kovaka et al. 2019) without reference annotations and using these parameters:

- Minimum isoform fraction (-f): 0.1
- Minimum reads per base pair coverage (-c): 2.5
- Fraction of bundle allowed to be covered by multi-hit reads (-M): 0.95

#### Library type: fr-firststrand

Transcriptomes from each condition were merged in “merge” mode of StringTie with a minimum input transcript per million (TPM) of 0.5 (-T 0.5). Expression levels were quantified using RNA-seq by expectation-maximization (version 1.3.1) (Li and Dewey 2011) with the -star option for alignments.

#### Processing of ONT cDNA sequencing data

ONT cDNA reads were first filtered by a minimum average read quality score ≥ 7 using NanoFilt (version 2.8.0) (De Coster and Rademakers 2023), then trimmed of adapter sequences and reoriented with Pychopper (https://github.com/epi2me-labs/pychopper). LoRDEC (version 0.9) (Salmela and Rivals 2014) was used to correct remaining base-calling errors in ONT reads, relying on 21-mers from matched-condition Illumina RNA-seq data. Long read–based transcriptome assembly was performed with FLAIR (version 1.5.1) (Tang et al. 2020) with the following minor modifications:

- Reads with ≥80% exonic overlap and an identical intron chain to assembled transcripts were considered full-length.
- Assembled transcripts supported by ≥5 full-length reads were retained.

Condition-specific transcriptomes were merged via an in-house script (Supplementary Fig. 2): single-exon transcripts with overlapping exons were merged into a single, longer transcript, whereas multiexon transcripts sharing the same intron chain had their alternative 5′ or 3′ ends merged if they were within 100 nt (5′) or 15 nt (3′). ONT reads were aligned to the reference genome using minimap2 (version 2.17-r941) (Li 2018) with parameters -MD -ax map-ont -N 10 -u f -a. Expression quantification employed IsoQuant (version 3.3.0) (Prjibelski et al. 2023) in -data_type nanopore mode.

#### Integration and classification of ONT- and Illumina-derived transcriptomes

As outlined in Supplementary Fig. 2, ONT single-exon transcripts with exonic overlap or ONT multiexon transcripts sharing intron chains with Illumina-derived transcriptomes were classified as “Tier 1.” Others were labeled “Tier 2.” Tier 2 Illumina transcripts were further refined via Co-Assembly of stranded and unstranded RNA-seq data Followed by End-correction pipeline (You et al. 2017), which updates exon junctions based on splicing reads and adjusts boundaries using cap analysis of gene expression (Brown et al. 2014) and 3P-seq data (Agarwal et al. 2018). Finally, Tier 1 ONT transcripts, Tier 2 ONT transcripts, and Tier 2 Illumina transcripts were aggregated into the initial hybrid transcriptome (Fig. 2a; Supplementary Fig. 2).

### Identification and elimination of ONT-specific intra-priming artifacts

To detect 5′ intra-priming artifacts from strand switching by the SSP, a local alignment was performed between the SSP and ±25 bp of the transcription start sites (TSS) using Bio.pairwise2 (align.localms, penalties: match=2, mismatch=-1, gap open=-10, gap extension=-0.1). For 3′ intra-priming artifacts by VN primer with oligo d(T), we measured nucleotide composition within 21 nt from −1 to +19 around transcript end sites (TES). Alignments scoring ≥25 for 5′ intra-priming or an adenosine frequency ≥0.8 for 3′ intra-priming flagged transcripts assembled from ONT as likely intra-priming artifacts. ONT-specific transcripts that did not exceed these thresholds but disrupted an annotated coding region were manually inspected and removed if they showed evidence of SSP or VN primer internal priming.

### Assessment of protein-coding potential

We evaluated the coding capacity of reference protein-coding, lncRNA, and novel lncRNA transcripts (≥5 count per million [CPM] for coding genes, ≥1 CPM for lncRNAs) using CPC (version cpc-0.9-r2) (Kong et al. 2007) and CPAT (version 3.0.4) (Wang et al. 2013).

### Tau specificity index

Tau specificity index was calculated to determine tissue- or condition-specific expression patterns (Yanai et al. 2005). In short, each gene's expression in a sample was divided by its maximum across all conditions, subtracted from 1, then summed across conditions and normalized by (*N* − 1), where *N* = 7 conditions


$$\mathrm{Tau}\;\mathrm{specificity}\;\mathrm{index}=\frac{\sum_{i=1}^{N}\;\left(1-\frac{\exp_{i}}{\max(\exp)}\right)}{N-1}$$

A higher value indicates stronger expression specificity.

### lncRNA classification according to their genomic context

The final set of lncRNAs was annotated by genomic location—genic vs intergenic—and further subdivided into antisense exonic, sense exonic, antisense intronic, sense intronic, intergenic, tandem array, convergent, or divergent types based on orientation and proximity to neighboring protein-coding genes (Supplementary Fig. 9b).

### Differentially expressed gene analysis between normal and wasp-infested conditions

Gene-level counts from ONT were analyzed via edgeR (version 3.40.2) (McCarthy et al. 2012) while accounting for RNA extraction and library preparation batches. A generalized linear model with quasi-likelihood was fit, and a quasi-likelihood F-test was used to identify differentially expressed gene (DEGs) between normal and infested lymph gland or blood at the same time points. lncRNAs with a fold change ≥2 and false discovery rate (FDR) ≤0.1 were considered differentially expressed.

### RT-qPCR method

Total RNA was extracted from the circulating hemocytes of 20 third-instar larvae at 120 h AEL. cDNA was synthesized using ReverTra Ace qPCR RT Master Mix (TOYOBO; FSQ-201) (Bong et al. 2024). qRT-PCR was performed by comparative Ct (ΔCt) method using SYBR Green Realtime PCR Master Mix (TOYOBO; TOQPK-201) relative to *Rp49* as a reference gene. Data were normalized to the *P1* primer expression under normal conditions using the ΔΔCt method (Hellemans et al. 2007). Primers used for the P1, P2, and P3 regions of the lncRNAs and the protein-coding transcript *CG14105* (Fig. 3c) are listed in Supplementary Table 2. For TSS validation, qPCR data were normalized to the reference gene *Rp49* to compare the relative abundance of different PCR targets within the sample. Relative expression levels were calculated using the 2^(-ΔCt) method. Primers for TSS validation are listed in Supplementary Table 2.

### Computational analysis of scRNA-seq data

Raw Drop-seq data from *Drosophila* larvae (GSE141273) (Cho et al. 2020) were processed using the Drop-seq tool (version 2.4.0, https://github.com/broadinstitute/Drop-seq/release/tag/v2.4.0). Metadata references for alignment were created via create_Drop-seq_reference_metadata.sh using default parameters, and alignment was carried out using Dropseq_alignment.sh. Cell barcodes were sorted by read counts and manually inspected via BAMTagHistogram to identify the “knee” threshold for cell selection (see Supplementary Table 3). A digital gene expression (DGE) matrix was generated using DigitalExpression and then merged across libraries.

The DGE matrix was analyzed in Seurat (version 5.0.3). Cell-type and subcluster labels from our previous work (Yoon et al. 2023) were used, and cells with ≥10% mitochondrial content were excluded, leaving 36,009 cells. Data were log-normalized and scaled, and principal components (PC) were selected using a JackStraw plot. Thirty PCs were used for downstream analyses. We employed Harmony (version 0.1.1) (Korsunsky et al. 2019) for integration of 30 single-cell libraries across 8 conditions. Clustering and dimension reduction were carried out with t-distributed stochastic neighbor embedding (t-SNE) and uniform manifold approximation and project (UMAP), respectively. Marker genes for each cell type were identified with FindAllMarkers (min.pct = 0.25, logfc.threshold = 0.75, test.use = “MAST”).

### Trajectory analysis of circulating hemocytes under normal and wasp-infested conditions

Drop-seq data of circulating hemocytes at 96 and 120 h AEL (normal vs wasp-infested) were used for pseudotime reconstruction in Monocle 3 (version 3_1.3.1) (Cao et al. 2019). Adipohemocytes and posterior signaling center (PSC) cells were excluded, given their lymph gland localization (Crozatier et al. 2004; Mandal et al. 2007; Cho et al. 2020). We followed the Monocle 3 pipeline (https://cole-trapnell-lab.github.io/monocle3/) with previous guidance (Cho et al. 2020):

1. **Data preprocessing**: *preprocess_cds(num_dim=45)*
2. **Removing unwanted sources of variation**: *align_cds* (library, total unique molecular identifiers [UMIs], mitochondrial fraction, cell cycle phases)
3. **Dimensional reduction**: *reduce_dimension(umap.min_dist=0.5, max_components=3)*
4. **Clustering**: *cluster_cells(resolution=0.00001)*
5. **Graph learning**: *learn_graph(close_loop* = *FALSE, use_partition* = *FALSE)*
6. **Cell ordering**: *order_cells* with the prohemocyte (PH) subcluster 1 set as the root (based on markers stg, polo, scra).

To isolate the LM lineage, we used *choose_graph_segments* between the PH subcluster 1 (PH1) and the LM endpoints. We applied *graph_test*(*q* < 0.01) to identify lncRNAs with spatial correlation along the trajectory, followed by manual selection of 6 lncRNAs with strong differential expression.

### Identification of co-expressed protein-coding genes with lncRNAs and GO term enrichment analysis

Because scRNA-seq exhibits dropouts and higher sparsity relative to bulk RNA-seq, we used scLink (Vivian Li and Li 2021) to build a sparse gene co-expression network. Read counts from circulating hemocytes along the LM lineage were library-normalized (10^6^ scale), and the top 8,000 most expressed genes were analyzed with *sclink_cor* under default parameters. Protein-coding genes with Pearson correlation coefficients ≥|0.4| relative to target lncRNAs were collected, and GO term enrichment (biological process, molecular function) was tested via *g:Profiler* (Kolberg et al. 2023), retaining the top 5 GO terms for positive and negative correlations (Fig. 5d).

### SABER-FISH

Signal amplification by exchanging fluorescence in situ hybridization (SABER-FISH) was performed to measure in situ lncRNA expression in larval hemocytes (Kishi et al. 2019), with minor adaptations. Twenty larvae were vortexed (1 min, 30 s) in 500 μL Phospate Buffered Saline (PBS) containing glass beads. Larvae were dissected in Teflon-printed slides (Immuno-Cell; 224072), 1 larvae per well, in cold Schneider's medium for 45 min at 4 °C. Cells were then fixed with 4% paraformaldehyde for 30 min at 25 °C, washed 3 times in PBSTw, and transitioned to Whyb buffer twice at 43 °C in a humid chamber. Hybridization with Hyb1/Probe proceeded at 43 °C for 16 h. Afterward, washes were done in Whyb and 2× Saline-Sodium Citrate buffet with Tween 20 (2 times each). Samples were returned to room temperature and washed twice with PBSTw. Fluorescent oligos were added to the samples and incubated at 37 °C for 3 h. Slides were mounted with VECTASHIELD with DAPI (Vector laboratories; H-1200-10). Three distinct probes targeting different regions of each lncRNA were used (see Supplementary Table 4).

### Generation of RNAi lines for target lncRNAs and *Drosophila* stocks

For lncRNA knockdown, *lncRNA:CR44948* and *lncRNA:CR44316* fragments were inserted into the WALIUM 22 vector (Drosophila Genomics Resource Center; 1473) (Hong et al. 2025). Transgenic lines were created by BestGene (BestGene Inc., CA, USA). RNA interference (RNAi) efficiency was verified (Supplementary Fig. 13b), and primer sequences are shown in Supplementary Table 5.

Fly stocks used in this study were maintained at 18 °C. Unless otherwise indicated, Gal4-UAS crosses were incubated at 25 °C and 70% humidity (Jung et al. 2026). *Srp-Gal4* (L.Waltzer), *HmlΔ-Gal4* (S.Sinenko), and Luciferase RNAi (BL31603) were used in this study. *Oregon R* was used as a wild-type (WT) strain. All fly stocks were maintained at 25 °C.

### Wasp infestation

Larvae were infested at 72 h AEL with *L. boulardi* (G486). Wasps were removed after 8 h of incubation and egg deposition was confirmed by observation of wasp eggs in the hemolymph during dissection.

### Identification of poly(A)-tailed reads and its clustering

Polyadenylation (poly[A])-tailed reads (PATRs) were detected as described in Moon et al. (2023), with minor modifications. We extracted Drop-seq reads from the 3 major cell types (LM, PH, and PM) and searched for ≥3 soft-clipped nucleotides at the 3′ end, with ≥80% adenosines in that region (or all adenosines if ≤5 nt in length). These were flagged as candidate PATRs. Their genomic locations were determined based on “XF” attributes from the BAM files generated by the Drop-seq pipeline. If PATRs were ≤50 nt from a transcript 3′ end, we categorized them as “3′ end” (Fig. 6b).

Because poly(A) cleavage is imprecise (Gruber et al. 2016), we clustered candidate PATRs near the 3′ ends following (Jan et al. 2011), with a 21-nt window around the most abundant position. All PATRs within that window were grouped into a single cluster, which was assigned to the nearest transcript 3′ end. For each transcript, PATRs in these clusters were deduplicated to quantify unique molecular identifies. To implement read parsing for soft clipping, we used the pysam Python module.

## Results

### High-confidence hybrid transcriptome assembly of *Drosophila* larval lymph gland and hemocytes

To systematically characterize coding and noncoding transcriptomes in *Drosophila* larval lymph grand and circulating hemocytes under normal and parasitic wIF at 96 and 120 h AEL, which correspond to 24 and 48 h after infestation, we employed a hybrid sequencing strategy (Fig. 1a). We combined ONT long-read cDNA sequencing (17 samples from 7 different conditions—4 for normal and 3 for infestation) with Illumina short-read RNA-seq (7 samples from 7 different conditions). Across 7 different conditions of lymph gland and circulating hemocytes (with N50 of ONT reads ranging from 700 to 1,300 nucleotides) (Supplementary Fig. 1a), the number of mapped reads was approximately 10 times higher in the Illumina data (∼3M reads for ONT; ∼ 35M reads for Illumina), yet mapped bases were comparable (∼3.3G bases for ONT; ∼3.5G bases for Illumina; Supplementary Fig. 1b and c; Supplementary Table 1). This indicates that both sequencing platforms cover a similar depth of RNA bases, but with different read length distributions.

**Fig. 1.**
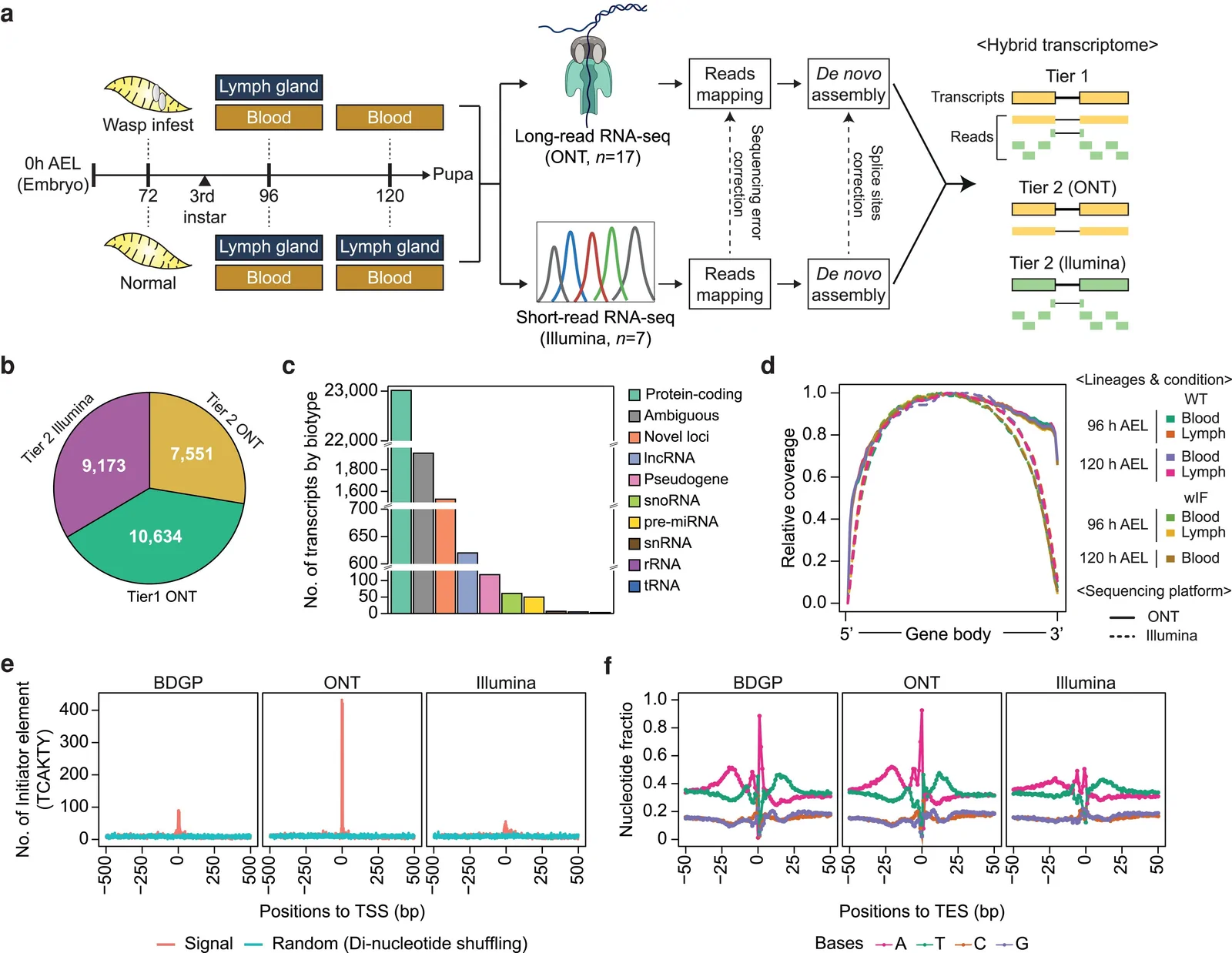
Hybrid transcriptome approach to identify to accurate structure of transcripts in *Drosophila* larval hemocytes. a) Experimental design and analytical workflow of ONT cDNA and Illumina sequencing for identification of transcripts in *Drosophila* larvae under normal or parasitic infestation by wasp. The number of sequencing samples exceeds the number of conditions in ONT data due to the inclusion of 2 to 3 biological replicates. h AEL, hour AEL. Created with bioRender (https://www.biorender.com). b) Number of transcripts identified across different tiers and sequencing platforms. c) Number of transcripts categorized by biotypes of overlapping genes. Ambiguous transcripts refer to those that exhibit intron retention or align to the opposite strand relative to reference annotation. d) Relative coverages of Illumina (dotted line) and ONT (solid line) sequencing data along the gene body are shown for each condition. WT, Wild-type; wIF, wIF. e) The frequencies of initiator elements (consensus sequence: TCAKTY) near the TSS of reference transcripts from BDGP and “Tier 1” transcripts from ONT and Illumina sequencing are shown. Random sequences were generated by dinucleotide shuffling of the original sequences. f) Nucleotide sequence composition near the TES of reference and “Tier 1” transcripts from ONT and Illumina sequencing.

ONT reads enabled precise detection of full-length transcripts and transcript boundaries, while Illumina reads provided highly accurate base calls for error correction and splice site confirmation. In the pipeline, erroneous ONT reads were corrected based on 21-mers and splice sites from Illumina data (Fig. 1a; Supplementary Fig. 2). We then assembled and merged transcripts separately from the ONT and Illumina platforms, using different criteria to consolidate overlapping transcripts. For ONT data, de novo assembly was performed with FLAIR, and resulting transcripts were merged if they shared the same intron chain (for multiexon transcripts) or showed exonic overlap (for single-exon transcripts), with shorter 5′/3′ ends subsumed into the longest transcript (100 nucleotide threshold for 5′ ends, 15 nucleotides for 3′ ends) (Supplementary Fig. 2 right). Illumina assemblies were merged using StringTie2 (Kovaka et al. 2019). When comparing the resulting assemblies from both platforms, we classified transcripts as “Tier 1” if they had exonic overlap (single exon) or identical intron chains (multiexon) across datasets, otherwise as “Tier 2” (Supplementary Fig. 2). For Tier 1, the ONT transcript was used as a representative structure. The initial hybrid transcriptome included ONT Tier 1 (*n* = 10,634 transcripts from approximately 7,000 gene loci), ONT Tier 2 (*n* = 9,173 transcripts from approximately 4,000 gene loci), and Illumina Tier 2 (*n* = 7,551 transcripts from approximately 5,000 loci) transcripts (Fig. 1b). When we classified the assembled transcripts according to their overlap with the reference gene annotation from the BDGP (version 6.22) (dos Santos et al. 2015), the majority overlapped with annotated protein-coding genes (*n* = 23,015), followed ambiguous transcripts exhibiting intron retention or opposite-strand matching (*n* = 1,950), novel loci (*n* = 1,529), and annotated lncRNAs (*n* = 620), among others (Fig. 1c).

Next, we evaluated the quality of the assembled transcripts. Coverage along the gene body showed that ONT reads exhibited improved coverage toward 3'end, while coverage at the 5′ end was less uniform and less clearly improved, though still slightly better than that observed for short-read dataset, which tended to drop sharply toward transcript termini (Fig. 1d) (Lahens et al. 2014). To independently confirm the authenticity of the identified termini, we examined promoter-associated motifs in *Drosophila*, including initiator motif (TCAKTY; Fig. 1e), and downstream promoter element (RGWYV; Supplementary Fig. 3 top) and TATA box motifs (TATAAA; Supplementary Fig. 3 bottom), as well as poly(A) cleavage signals (Fig. 1f). The known promoter-associated elements and poly(A) cleavage signals were significantly enriched at the 5′ and 3′ boundaries of ONT-derived transcripts. This enrichment also exceeded that observed in the reference annotations (particularly for transcription end sites, TES), highlighting the utility of ONT data in capturing bona fide transcript boundaries. Taken together, our hybrid transcriptome assembly of long- and short-read sequencing data resulted in high-confidence coding and noncoding gene annotations with accurate 5′ and 3′ boundaries.

### Removal of intra-priming artifacts reveals alternative or novel coding and noncoding transcripts

We next quantified gene- and isoform-level expression across all long-read datasets (17 ONT long-read libraries) using IsoQuant software (Prjibelski et al. 2023). PC analysis (PCA, Supplementary Fig. 4a) based on normalized expression showed that samples cluster by lineage (circulating blood or lymph gland) and condition (normal or wasp-infested). Across 7 conditions, gene expression levels from ONT were highly correlated with those from Illumina (Pearson correlation coefficients, *R*, ranging from 0.74 to 0.93), while correlations at the isoform level were slightly lower (*R* ranging from 0.62 to 0.78) (Supplementary Fig. 4b). Known markers such as LM-specific genes (e.g. *atilla* and *PPO3*) at 120 h with infestation (Crozatier et al. 2004) and PSC genes higher in lymph gland (Hultmark and Ando 2022) confirmed the biological relevance of each dataset (Supplementary Fig. 5a and b).

Despite these overall correlations, some isoforms appeared to be discordantly expressed between the ONT and Illumina data. Closer inspection after expression-based filtering (Fig. 2a) revealed clusters of ONT reads that anomalously accumulated at 5′ or 3′ segments of gene bodies, prompting us to investigate potential 5′, 3′ intra-priming artifacts introduced during 5′ strand switching or 3′ poly(A) selection steps (Fig. 2b, top). Specifically, sequences highly similar to the SSP and stretches of internal poly(A) were found to create false TSS or TES. The SSP is used during cDNA synthesis to enable template switching at the 5′ end of transcripts. However, nonspecific annealing or mis-priming can introduce artifactual sequences that mimic genuine transcription start sites. Similarly, poly(A) intra-priming occurs when oligo(dT) primers anneal to internal A-rich regions within transcripts rather than true poly(A) tails, leading to truncated cDNAs that falsely appear as transcription end sites.

**Fig. 2.**
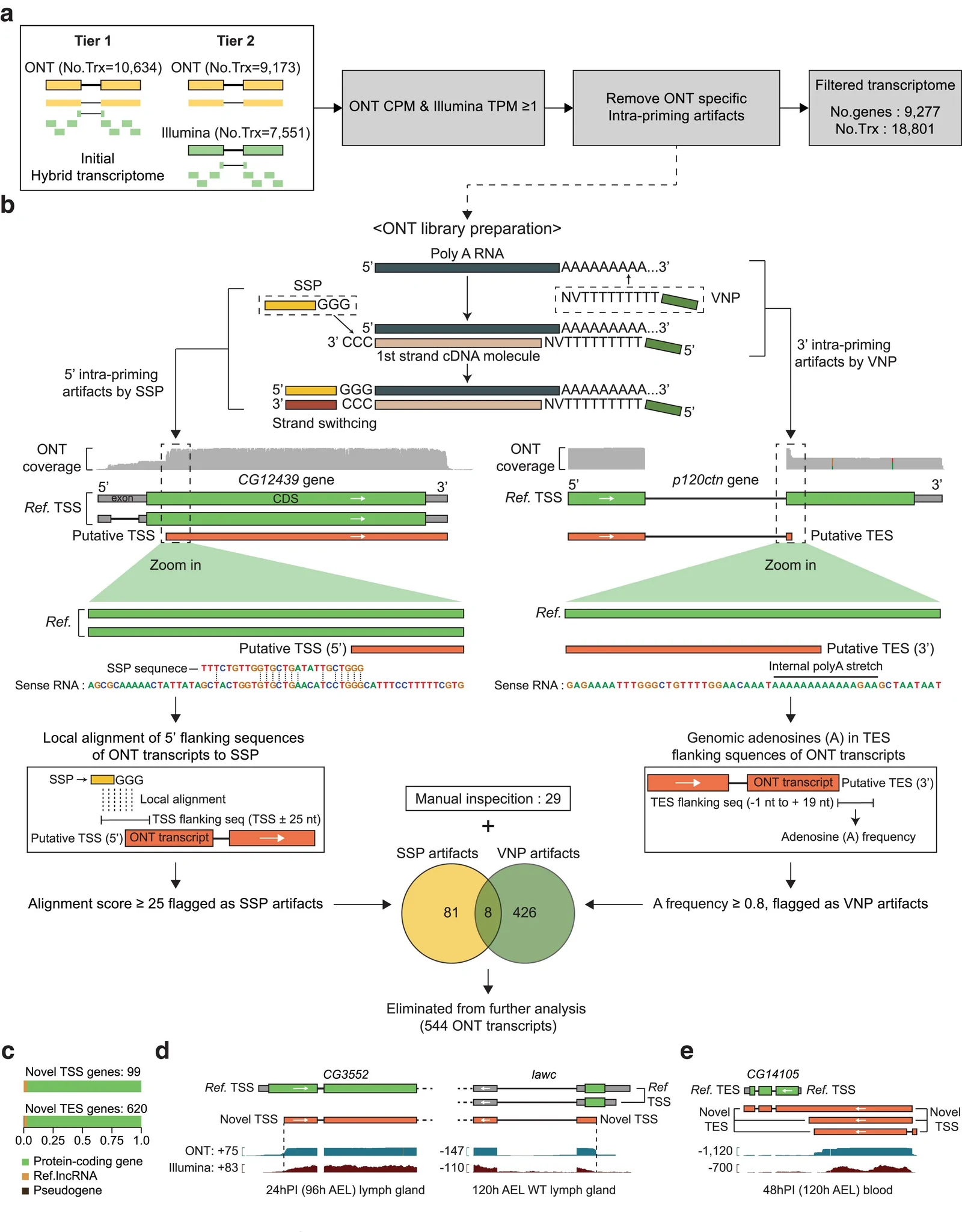
Identification and elimination of ONT-specific intra-priming artifacts and discovery of potential bifunctional genes in *Drosophila* larval hemocytes. a) Overview of the transcriptome filtering steps to obtain a high-confidence set of transcripts. The initial hybrid transcriptome (produced at the end of hybrid transcriptome assembly pipeline specified in Supplementary Fig. 2, *n* = 27,358), consisting of ONT “Tier 1” and “Tier 2” and Illumina “Tier 2” transcripts, was initially filtered using RNA expression levels from both sequencing data (CPM for ONT and transcript per million; TPM for Illumina data). Subsequently, ONT-specific intra-priming artifacts were identified and eliminated, resulting in a filtered transcriptome (*n* = 18,801). Trx, transcripts. b) Summary of detection and elimination of ONT-specific intra-priming artifacts. (Top) Schematics of synthesis of first strand cDNA molecule and strand switching reaction during ONT cDNA library preparation are depicted. (Middle) Examples of intra-priming artifacts arising from SSP at the 5′ end and VN primer with oligo(dT) at 3′ end of gene bodies are shown. (Middle left) In the *CG12439* gene locus, sequences near the putative TSS of newly assembled transcript exhibit high similarity to the SSP sequence. (Middle right) In *p120ctn* gene locus, sequences near the putative TES of newly identified transcript contain internal poly(A) stretch. (Bottom left) For a systematic detection of ONT-specific intra-priming artifacts, local alignments were performed between transcription start sites flanking sequences (transcription start sites ± 25 nt) and SSP sequences for 5′ intra-priming events. Alignments with scores ≥25 were identified as potential artifacts. (Bottom right) For 3′ intra-priming events, base compositions near transcription end sites flanking sequences (1 nt upstream to 19 nt downstream of the transcription end sites) were examined and sequences with an adenosine frequency ≥0.8 were considered potential artifacts (bottom middle). As a result, a total of 515 intra-priming candidates, along with 29 manually handpicked candidates, were eliminated from further analysis. c) Number of genes with novel TSS (*n* = 99) and TES (*n* = 620), along with their biotype composition. d and e) Schematic illustration candidate bifunctional genes in *Drosophila* larvae. d) Newly identified transcripts with novel transcription start sites in *CG3552* (left) and *lawc* gene loci (right) with truncated 5′ ends that disrupt the CDS, potentially leading to loss of protein-coding ability. e) Three newly identified transcripts with either novel transcription start sites (second from the top) or both novel transcription start and end sites (third and fourth from the top) in or near the *CG14015* gene locus. The third and fourth transcripts were predicted to lack proper CDS region in silico (see “Materials and Methods”). b, d, and e) Green box, CDS region; gray box, exon; arrow indicates direction of transcription.

To systematically assess artefactual transcripts arising from these mis-priming events, we first flagged transcripts with perturbed coding sequences. Local alignment and adenosine count of the flagged transcripts confirmed similarity to the SSP at the 5′ ends of 30 transcripts (alignment score ≥ 25; Supplementary Fig. 6a) and internal poly(A)-mediated artifacts at the 3′ ends of 66 transcripts (the fraction of adenines in a specific 21-nucleotide window of the transcript ≥ 0.8; Supplementary Fig. 6b). Applying the same criteria to all ONT-derived transcripts, we systematically identified 515 transcripts plus 29 additional manually curated cases as intra-priming artifacts, all of which were eliminated from further analysis (Fig. 2b, bottom).

After removing low expressed transcripts and intra-priming artifacts, 18,801 transcripts from 9,277 genes remained (Fig. 2a; Supplementary Fig. 7), which were then compared to the BDGP reference gene model (version 6.22) using GFFcompare (Pertea and Pertea 2020) (Supplementary Fig. 7). Of these, 12,702 transcripts showed an exact intron chain match to reference transcripts (Supplementary Fig. 7, YES part). Among them, we detected 679 transcripts with novel boundaries (99 for novel transcription start sites, 620 for novel transcription end sites, and 40 with both novel transcription start and end sites)—differing from the annotated 5′ or 3′ ends by more than 100 and 15 nucleotides, respectively (Fig. 2c; Supplementary Fig. 7, bottom left)—indicating widespread unannotated transcription start and termination sites of protein-coding and lncRNA genes in *Drosophila* larvae. Besides transcripts with novel 5′ or 3′ ends, the remaining 6,288 transcripts were assessed relative to reference lncRNA annotation (Supplementary Fig. 7, NO part). Among these, transcripts that either overlap with exonic regions of reference lncRNAs or originate from novel loci were identified and subsequently evaluated for protein-coding potential using *in silico* approaches. As a result, 93 new isoforms (including 74 lncRNA and 19 ambiguous isoforms) within 59 known lncRNA loci and 408 new transcripts (including 275 lncRNA, 72 ambiguous, and 61 putative-coding transcripts) within 245 novel loci were newly identified (Supplementary Fig. 7, bottom right).

Among these newly identified transcripts and isoforms, 101 of them exhibited specific expression pattern under wIF, suggesting the presence of condition-specific regulation. Taken together, these results confirm that the hybrid pipeline yields a high-confidence transcriptome, expands the repertoire of novel coding and noncoding isoforms, and highlights the importance of stringent artifact removal for accurate analysis of coding and noncoding RNAs.

### Alternative transcription start sites of coding genes create novel noncoding isoforms

Among transcripts with a partial intron chain match or partial exon overlap with the protein-coding genes, we identified those in which the annotated coding sequence (CDS) region was disrupted, resulting in noncoding isoforms truncated at the 5′ or 3′ end of the CDS that were not associated with ONT-specific intra-priming events (Fig. 2d and e; Supplementary Fig. 8a to e). For *CG3552* and *lawc*, newly discovered transcription start sites were supported by reads from both short-read and long-read sequencing data, and examination of the genomic sequences confirmed that these transcription start sites were unrelated to 5′ intra-priming by SSP (Fig. 2d; Supplementary Fig. 8a and b). Three additional genes, *CG7882*, *Cyp1*, and *rush*, also contained novel transcription start sites–derived noncoding isoforms (Supplementary Fig. 8c to e). Of these, *Cyp1* and *rush* simultaneously expressed both annotated CDS and noncoding isoforms, whereas *CG7882* predominantly expressed novel noncoding isoforms in all conditions sampled. Moreover, novel transcription start sites of *CG3552*, *Cyp1*, and *rush* were in vivo validated (Supplementary Fig. 8f). These results suggest that both mis-annotations in the current BDGP reference and true bifunctional genes (Nam et al. 2016) may exist in *Drosophila*. Furthermore, in *CG14105*, several transcripts with novel transcription start or end sites, or both were supported by reads from both sequencing platforms (Fig. 2e). Transcripts harboring these novel transcription end sites appear to have lost the protein-coding region, retaining only noncoding regions. It would be interesting to elucidate the functional roles of these putative bifunctional genes by alternative transcription end sites.

### *Drosophila* larvae lncRNAs under normal and parasitic wIF

In our dataset, lncRNAs exhibited greater expression specificity—defined as more restricted expression across cell types or conditions—than protein-coding genes (tau specificity index; Supplementary Fig. 9a) (Yanai et al. 2005), as previously reported (Cabili et al. 2011; Derrien et al. 2012; Pauli et al. 2012). We further investigated how lncRNA expression differs between uninfested and wasp-infested hemocytes. We quantified the expression of annotated and newly identified lncRNAs, categorized by strand and position relative to neighboring protein-coding genes (Supplementary Fig. 9b), in lymph gland and circulating hemocytes at 96 and 120 h AEL after wIF at 72 h AEL. In total, 117 lncRNAs, including 52 novel transcripts, were observed to be differentially expressed (fold change ≥ 2 and FDR ≤ 0.1 in edgeR [McCarthy et al. 2012] using ONT-derived gene counts) between matched tissues and time points (Fig. 3a). Approximately half of the lncRNAs were strongly upregulated at 120 h AEL (48 h post-infestation) in circulating hemocytes, suggesting their involvement in immune response to wasp parasitism. Notably, the remaining lncRNAs showed highly stage-specific expression in uninfested larvae (Fig. 3a), underscoring the developmental regulation of lncRNA transcription.

**Fig. 3.**
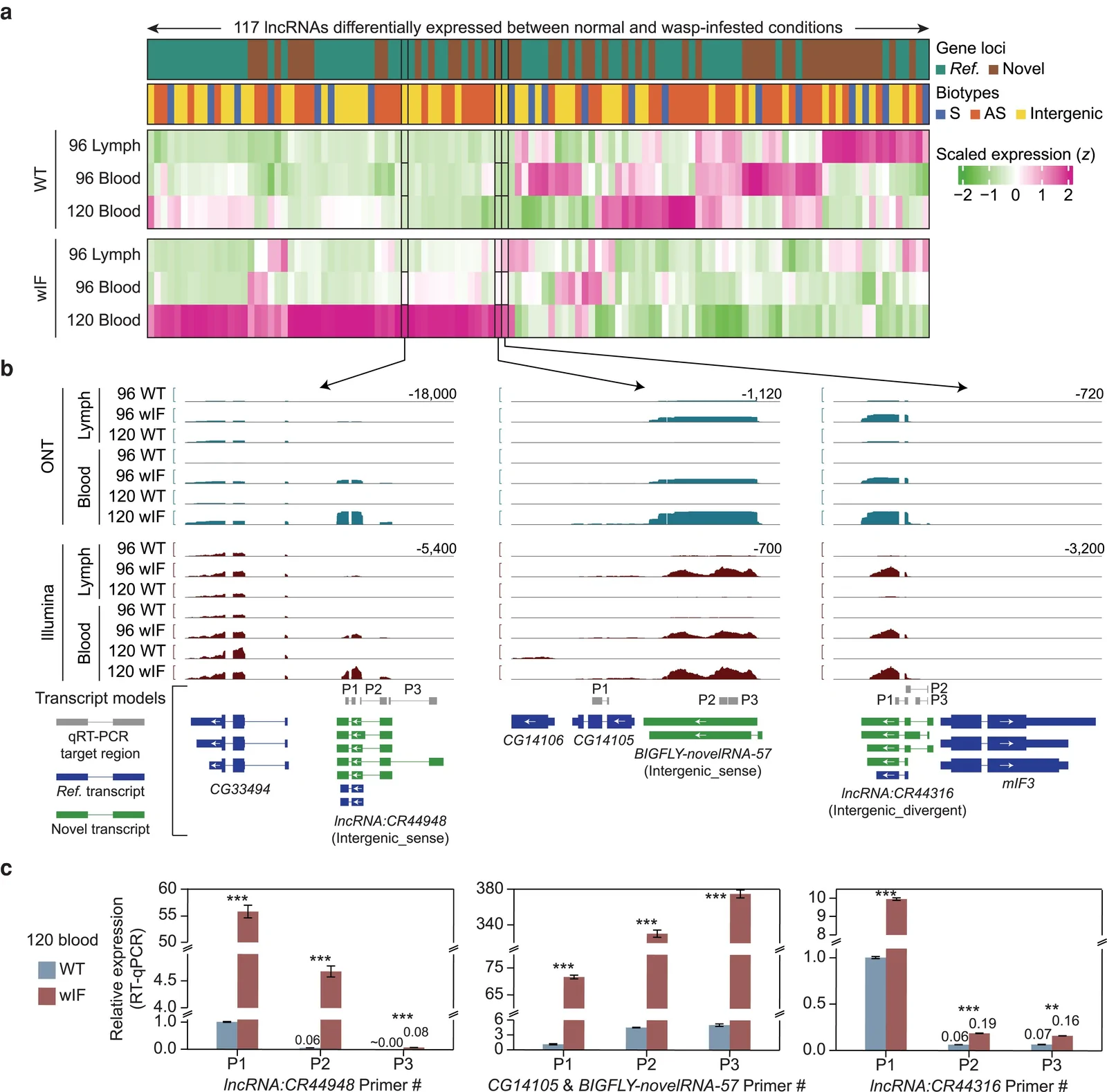
A substantial number of lncRNAs are strongly induced in *Drosophila* larval hemocytes after wIF. a) Normalized and scaled expression levels of 117 lncRNAs across the 6 samples shown in the panel, which are significantly and differentially expressed between normal and infested conditions within the same tissue and time point (see “Materials and Methods”). S, sense (both intronic and exonic) genic lncRNAs; AS, antisense (both intronic and exonic) genic lncRNAs. b) Genomic regions and read coverages of 3 intergenic lncRNAs that are strongly expressed in circulating hemocytes of *Drosophila* larvae at 48 h postinfection compared to normal condition. The direction of transcript is indicated by white arrow and only the sequencing coverages matching the strands of lncRNAs of interest are shown. c) For each candidate lncRNA highly expressed under wIF, 3 RT-qPCR primers were designed and utilized to quantify expression levels of unique exon junctions or exonic region of lncRNAs or neighboring transcript in normal or infested circulating blood at 120 h AEL. Ct values were normalized to expression level of P1 in normal circulating blood at 120 h AEL for each genomic region (see “Materials and Methods” for details). WT, wild-type; wIF, wasp infestation. **P < 0.01, ***P < 0.001 (2-sided, unpaired Student's *t*-test).

Focusing on 3 intergenic lncRNAs that are highly upregulated under wIF (Supplementary Fig. 9c), we validated lncRNA:CR44948 (Fig. 3b, left), BIGFLY-novelRNA-57 (Fig. 3b, middle), and lncRNA:CR44316 (Fig. 3b, right). Our hybrid assembly revealed 5 novel isoforms for lncRNA:CR44948 and 4 for lncRNA:CR44316. BIGFLY-novelRNA-57, previously classified as one of the CG14105 isoforms (Fig. 2e), was redefined as an independent gene with 2 isoforms based on its noncoding features and expression patterns (Fig. 3b). RT-qPCR using 3 different primer sets confirmed significant upregulation of these lncRNA isoforms in circulating hemocytes at 120 h AEL (48 h PI) under wasp-infested conditions, validating the presence of novel splicing isoforms of 3 intergenic lncRNA genes (Fig. 3c). Yet, the biological significance of these newly identified lncRNAs in relation to their expression levels remains to be determined in future studies.

Taken together, these findings expand the repertoire of lncRNAs in *Drosophila* larvae and reveal dynamic changes in noncoding transcript expression across developmental stages and immune conditions.

### Enhanced annotation of transcription end sites improves scRNA-seq analysis of *Drosophila* larvae

To facilitate single-cell analyses of both coding and noncoding transcripts, we integrated the results of our hybrid transcriptome assembly—including novel protein-coding isoforms and novel lncRNAs—into the existing *Drosophila* reference gene annotation (BDGP version 6.22), creating an updated annotation referred to as BDGA version 1.0 (Supplementary data 1). Our group previously described the transcriptomic profiles of lymph glands and circulating hemocytes from *Drosophila* larvae at 72, 96, and 120 h AEL under normal and wasp-infested conditions at single-cell resolution (Cho et al. 2020; Tattikota et al. 2020; Yoon et al. 2023). In total, we reanalyzed 30 independent single-cell libraries from 8 conditions, spanning 3 larval stages (72, 96, and 120 h AEL) under WT and wasp-infested conditions, using both the original reference (BDGP version 6.22) and our updated BDGA version 1.0 (Fig. 4a and b). Compared to the original reference, our updated annotation captured over 1 million additional reads in the single-cell analysis (Fig. 4a). Furthermore, transcripts with newly identified transcription start and end sites contained a higher number of UMIs than transcripts without redefined boundaries (Fig. 4b). For example, 2 novel isoforms with alternative transcription end sites at the CG33494 locus increased read coverage by approximately 17% (∼38 K reads) (Fig. 4c). The read peaks for these isoforms aligned more precisely with the novel transcription end sites (dotted box in Fig. 4c) than with the reference transcription end sites, validating our newly discovered transcription end sites. Taken together, these results confirm that our updated gene annotation improves the accuracy of single-cell quantification.

**Fig. 4.**
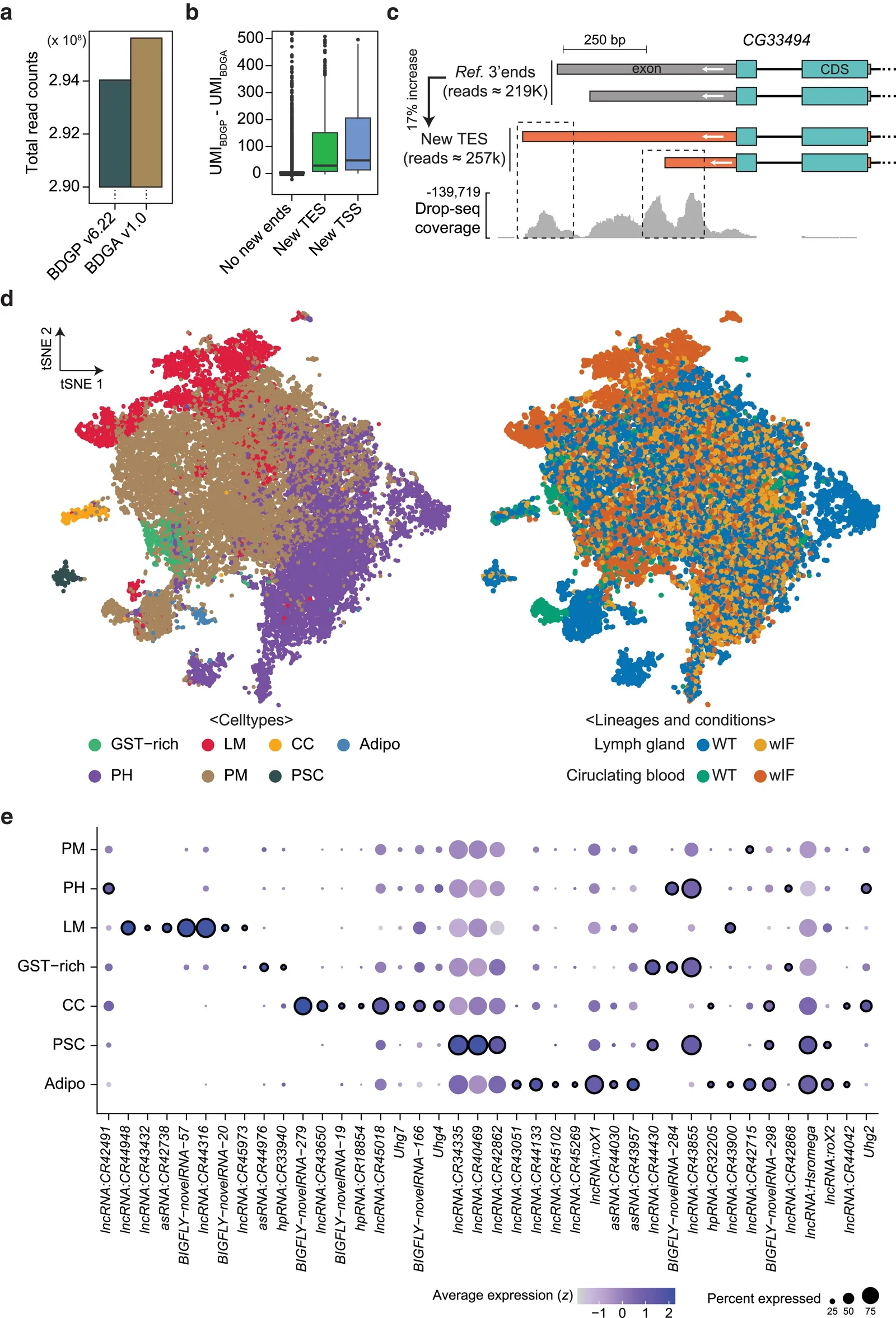
Improved single-cell analysis using updated gene model and identification of cell type–specific lncRNA markers, particularly in the LM population. a) Comparison of the total number of reads utilized in Drop-seq analysis using BDGP v6.22 (reference) and BDGA v1.0 (updated) gene annotations. b) Distribution of difference in the number of detected UMIs between the BDGP v6.22 and our BDGA v.10 gene annotation (UMI_BDGP_ − UMI_BDGA_) across different gene groups. No new ends, genes with no new transcript ends discovered; New TES, genes with new transcription end sites; New TSS, genes with new transcription start sites. c) Schematic representation of *CG33494* gene locus where the identification of new transcription end sites led to approximately 17% increase in unique molecular identifiers detection. Drop-seq peaks near the new transcription end sites are indicated in dotted boxes. d) (Left) A t-SNE plot showing 2D projection of 7 major cell types identified in the scRNA-seq dataset (*n* = 36,009). PH, prohemocytes; LM, lamellocytes; PM, plasmatocytes; CC, crystal cells; PSC, posterior signaling center; Adipo, adipohemocytes. (Right) The same t-SNE plot depicting cells of different conditions in *Drosophila* larvae from 72 to 120 h AEL. WT, wild-type; wIF, wasp infestation. e) Expression levels of lncRNA marker genes identified in 7 major cell types. Dot color indicates average scaled expression levels, and dot size displays the percentage of cells that express the corresponding lncRNA gene. For each cell type, lncRNA genes that are expressed in less than 10% of the cell population are not plotted.

Using this updated annotation for single-cell data integration reproduced the key findings of our previous single-cell study (Yoon et al. 2023). After discarding cells with mitochondrial content above 10% (leaving 36,009 cells; Supplementary Fig. 10a), the resulting dataset reflected typical *Drosophila* hemocyte populations under both WT and wIF conditions, including PMs, PHs, CCs, and LMs (Fig. 4d; Supplementary Fig. 10b and c). Consistent with previous reports (Anderl et al. 2016; Leitao et al. 2020; Tattikota et al. 2020), LMs predominantly appeared under infested conditions. The expression patterns of well-characterized cell type–specific protein-coding markers in larval hemocytes were also verified (Supplementary Fig. 11).

We next examined both reference and novel lncRNAs that are specifically expressed in certain hemocyte subpopulations (Fig. 4e). Notably, several previously characterized LM-enriched lncRNAs—lncRNA:CR44948, *lncRNA:CR43432*, asRNA:CR42738, and lncRNA:CR44316—were again identified as LM markers in our analysis (Cho et al. 2020; Leitao et al. 2020; Tattikota et al. 2020). In addition to these reference lncRNAs, we discovered novel lncRNAs, including *BIGFLY-novelRNA-57* and *BIFLY-novelRNA-20*, that are enriched in LMs and CCs, respectively. Furthermore, *lncRNA:CR44948*, *lncRNA:CR43432, lncRNA:CR42738, lncRNA:CR44316*, and *lncRNA:CR45973*—all of which increased during parasitic wIF (Fig. 4e; Supplementary Fig. 9c)—also mapped specifically to LMs. This observation is consistent with the specialized role of LMs in immune responses against parasitic wIF. Taken together, these results suggest potential regulatory functions for these noncoding RNAs in hemocyte development and differentiation, particularly within LM and CC lineages essential for anti-wasp parasitism (Banerjee et al. 2019).

### Discovering noncoding landscape along developmental trajectory of circulating hemocyte

LMs serve as critical effector cells in the immune defense against parasitic wasps in *Drosophila* larvae (Dudzic et al. 2015). During wIF, hemocytes proliferate, and LMs differentiate from both circulating hemocytes and lymph gland PH (Honti et al. 2014; Cho et al. 2020). To investigate the developmental origins of LMs, we reconstructed the developmental trajectories of circulating hemocytes under normal and wasp-infested condition using Monocle3 (Cao et al. 2019). Consistent with the previous work on hematopoietic lineages in the *Drosophila* larval lymph gland (Cho et al. 2020), we designated PH1 as the root cell based on the expression of progenitor cell markers and cell cycle genes, including *stg*, *polo*, and *scra* (Supplementary Fig. 12a). However, PH1 hemocytes in circulation may be distinguishable from PH residing in the lymph gland, and future studies will be required to fully characterize these cells.

Pseudotime reconstruction of circulating hemocytes revealed multiple trajectories originating from PH1 and leading to PMs or LMs under normal and wasp-infested conditions (Fig. 5a and b; Supplementary Fig. 12b). We focused on the LM lineage trajectory and searched for lncRNAs that showed statistically significant structured correlation within this lineage. Using the graph_test function in Monocle3 with a strict threshold (*q* < 0.01) (Cao et al. 2019), we identified 11 lncRNAs correlated with pseudotime along the LM lineage, of which 6 representative lncRNAs are shown in Fig. 5c. Among them, 2 lncRNAs—*lncRNA:CR43855* and *BIGFLY-novelRNA-164*—showed decreased expression over pseudotime, whereas 4 lncRNAs—*lncRNA:CR44316*, *BIGFLY-novelRNA-57*, *lncRNA:CR44948*, and *lncRNA:CR43432*—showed a sequential increase in expression upon hemocyte differentiation into LMs. This observation is consistent with our previous detection of these lncRNAs as LM markers (Fig. 4e).

**Fig. 5.**
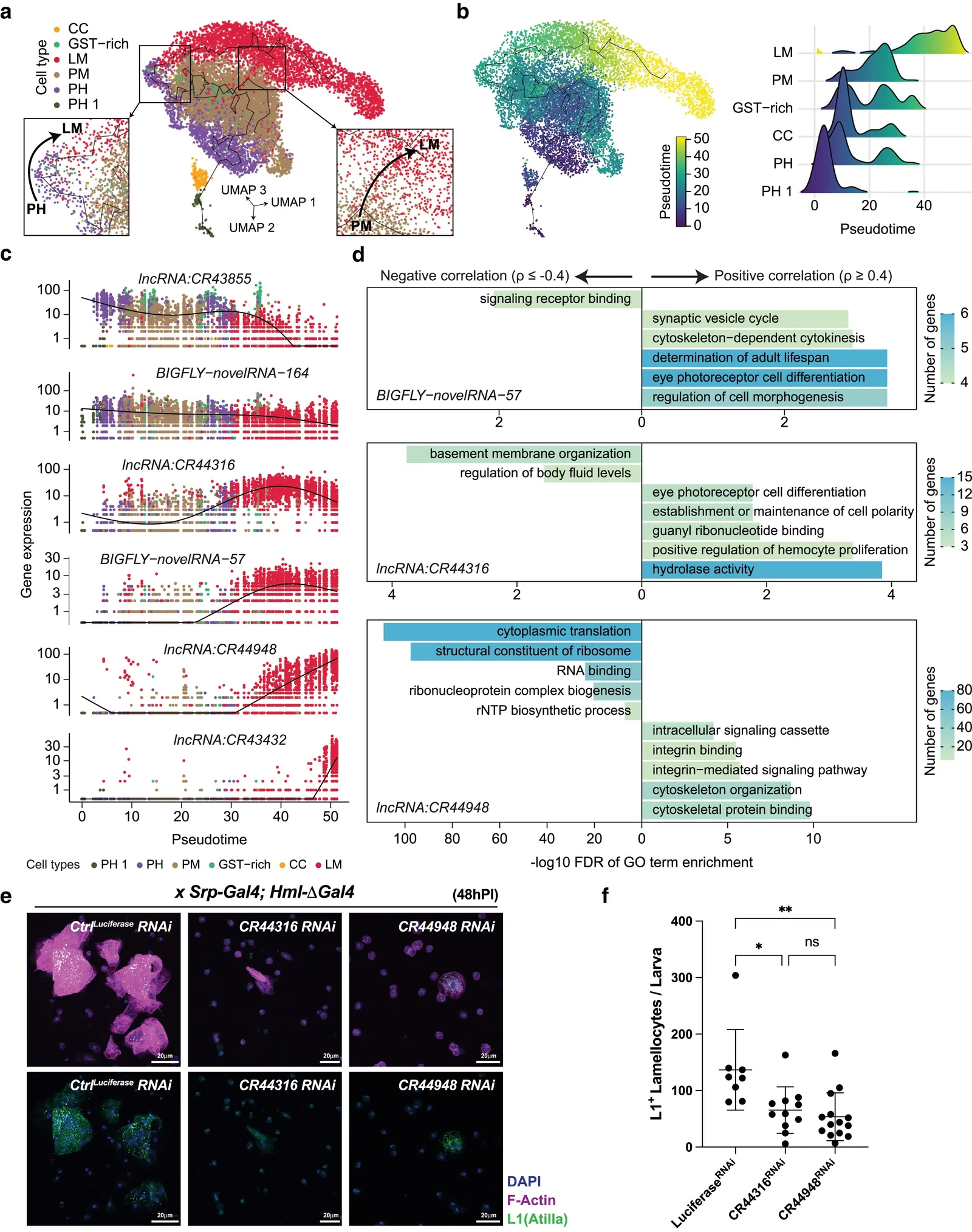
Identification of lncRNAs with structured correlation within the *Drosophila* larval hemocyte lineage and their biological functions. a) A 3D UMAP trajectory landscape of *Drosophila* larval hemocytes from circulating blood (left), along with additional representation of trajectory over calculated pseudotime using Monocle 3 (right) under normal and wasp-infested conditions. b) Relative densities of the circulating hemocytes under normal and wasp-infested conditions along pseudotime (left). PH1 subcluster is separated from other PH population for higher resolution. Relative proportion of cell phases in each cell type is also depicted (right). c) Expression levels of lncRNAs with structured correlation within the LM lineage are shown along pseudotime, with each dot representing a single cell of a specific cell type. d) GO enrichment analysis of protein-coding genes that are significantly correlated with *BIGFLY-novelRNA-57, lncRNA:CR44316*, and *lncRNA:CR44948* within circulating hemocytes of the LM lineage. Significantly enriched GO terms for negatively (ρ ≤ −0.4) and positively (ρ ≥ 0.4) correlated genes are shown as bar plots on the left and right, respectively. e) Knockdown of *lncRNA:CR44948* and *lncRNA:CR44316* in pan-hemocytes reduced the formation of LMs after 48 h after wIF. Knockdown of lncRNAs in pan-hemocyte were performed and LMs were detected by L1 (Atilla) staining (green) with F-Actin (magenta) (*Srp-Gal4, HmlΔ-Gal4; UAS-Luciferase RNAi, UAS-CR44948 RNAi* or *UAS-CR44316 RNAi*). f) Quantification of L1 + LMs from (e). Tukey's multiple comparison test was used for statistics. ns; not significant, *; *P <* 0.05. **; *P <* 0.005.

To validate the expression of these lncRNAs in *Drosophila* larval hemocytes *in vivo*, we performed the SABER-FISH (Kishi et al. 2019), a multiplexed approach for sensitive in situ detection of RNA molecules. Consistent with our transcriptomic analysis, the signals for *lncRNA:CR44948*, *lncRNA:CR44316*, and *BIGFLY-novelRNA-57* were highly elevated in larval hemocytes following wIF (Supplementary Fig. 13a). These results confirm that a specific subset of lncRNAs is upregulated in larval hemocytes in response to parasitic wasp challenge.

### The functional role of wIF-induced lncRNAs in LMs

As *Drosophila* lncRNAs often exhibit low sequence conservation with those in human and mouse genomes, it is challenging to pinpoint their functional relevance (Kopp and Mendell 2018). We therefore adopted a guilt-by-association strategy to predict the roles lncRNAs by examining their correlation with protein-coding genes in circulating hemocytes aligned along the LM lineage. Using scLink (Vivian Li and Li 2021) to construct a sparse gene co-expression network, we performed gene ontology (GO) enrichment analysis on protein-coding genes that showed significant positive or negative correlations (Pearson ρ ≤ −0.4 or ≥0.4) with 3 LM-specific lncRNA: *lncRNA:CR44948*, *BIGFLY-novelRNA-57*, and *lncRNA:CR44316* (Fig. 5d). Genes inversely correlated with lncRNAs were enriched for functions in signaling receptor binding, basement membrane organization, cytoplasmic translation, and ribosome components. In particular, *lncRNA:CR44948* showed a strong negative association with protein translation processes, implying a regulatory role in protein translation. Conversely, positively correlated GO terms included cell differentiation, cytoskeleton, cellular signaling, and proliferation, suggesting that these lncRNAs contribute to hemocyte differentiation, proliferation, and maintenance. For example, *BIGFLY-novelRNA-57* and *lncRNA:CR44316* were associated with cell morphogenesis and positive regulation of hemocyte proliferation, respectively, suggesting its functions in hemocyte expansion during immune responses.

To validate the roles of *lncRNA:CR44316* and *lncRNA:CR44948* in LM differentiation and hemocyte proliferation, we generated RNAi lines targeting these lncRNAs using the GAL4-UAS system (Brand and Perrimon 1993). Knockdown of *lncRNA:CR44948* or *lncRNA:CR44316* in pan-hemocytes (*Srp-Gal4; HmlΔ-GAL4 UAS-lncRNA:CR44948 RNAi* or *Srp-Gal4; HmlΔ-GAL4 UAS-lncRNA:CR44316 RNAi*) significantly attenuated LM differentiation (Fig. 5e and f; Supplementary Fig. 13b), demonstrating that these lncRNAs are essential for LM development during wIF in *Drosophila* larvae.

In summary, using our updated gene annotation, we reconstructed the trajectories of *Drosophila* larval circulating hemocytes under normal and parasitic wasp–infested conditions and uncovered several lncRNA markers that are differentially expressed along the LM lineages. Both in silico functional predictions and in vivo experimental validations highlight that these lncRNAs are critical regulators of LM differentiation and proliferation. These findings position them as promising targets for uncovering novel noncoding regulatory pathways in *Drosophila* immune responses.

### Detection of novel alternative poly(A) events across major hemocyte cell types

APA is a critical mechanism of gene regulation that contributes to transcriptomic diversity by generating variable 3′ UTRs and transcription end sites. Using our hybrid transcriptome assembly, we previously identified 620 transcripts with novel alternative 3′ ends in *Drosophila* larvae (Supplementary Fig. 7). To assess these newly discovered transcription end sites at the single-cell resolution, we employed identify and utilize PATRs from scRNA-seq data. These PATRs contain poly(A) tails and map to the most distal position of the gene locus, providing direct evidence for polyadenylation sites (Li et al. 2021; Moon et al. 2023). Although they are rarely captured in single-cell sequencing technologies, these reads are located near the 3′ ends of transcripts.

We extracted scRNA-seq reads with ≥3 soft-clipped nucleotides and ≥80% adenosines in the soft-clipped at the 3′ end of genes (Fig. 6a; Supplementary Fig. 14a). Soft-clipped nucleotides refer to portions of a sequencing read that do not align to the reference genome and are therefore clipped by the aligner, often reflecting untemplated sequences such as poly(A) tails or adapter-derived sequences at transcript ends. Due to their low abundance, reads from 3 major cell types (LMs, PHs, and PMs) were pooled for analysis. These candidate PATRs s were then categorized based on their genomic location. We found that 57% to 65% were located within 50 nt of known or novel transcript 3′ ends, with the remainder located within coding, UTR, or intergenic regions (Fig. 6b).

**Fig. 6.**
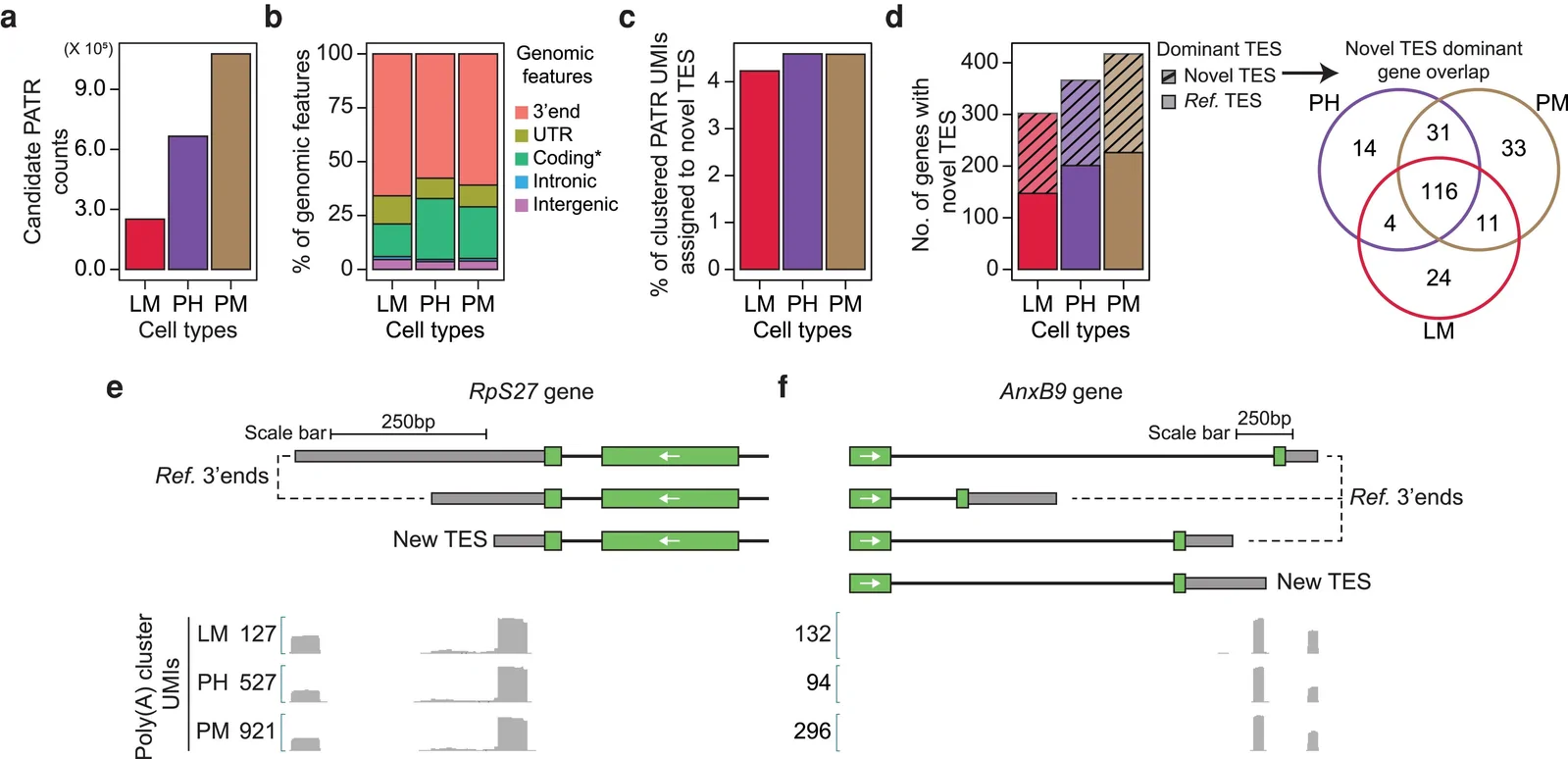
Evidence of newly identified transcription end sites from PATRs in drop-seq data. a) Number of candidate PATRs detected in single-cell RNA-seq data across 3 major cell types (LM, PH, and PM). b) Percentage of genomic features where candidate PATRs are located across 3 major cell types. Note that “Coding” includes candidate PATRs mapped to exonic regions in noncoding RNAs. c) Percentage of unique molecular identifiers assigned to newly identified transcription end sites for each cell type. Clustering of PATRs and deduplication were conducted as in Supplementary Fig. 13b, albeit performed separately for each cell type. d) (Left) Number of genes with novel transcription end sites identified through our hybrid transcriptome assembly approach, where at least 1 poly(A) site is supported by clustered PATRs, is shown for each cell type. Genes where novel transcription end sites represent dominant poly(A) sites are shaded. (Right) Overlap of genes with novel transcription end sites as dominant poly(A) sites between cell types are shown. e and f) Schematic representations and coverages of PATR cluster and gene locus with newly identified transcription end sites. e) The number of reads in PATR cluster is consistently higher in new transcription end sites across cell types at the *RpS27* gene locus. f) The transcript with new transcription end site is more highly expressed in PH at the *AnxB9* gene locus, indicated by the number of reads in PATR clusters.

Because poly(A) cleavage sites typically lack single-nucleotide precision (Jan et al. 2011; Gruber et al. 2016), we clustered candidate PATRs s near the 3′ ends of transcripts. This clustering was performed across all 3 major cell types, combining PATRs s within ±10 nt of the most abundant position into a single cluster. Each PATRs cluster was then assigned to its nearest transcript 3′ end. Among 7,664 transcripts originating from 4,410 gene and supported by ≥2 UMIs (see “Materials and Methods”), the number of UMIs per transcript ranged from 2 to 37,362 (Supplementary Fig. 14b). Most of these polyadenylation-supported genes were protein-coding (*n* = 6,110), along with the annotated lncRNAs (*n* = 126) and novel lncRNAs (*n* = 71).

To assess how thoroughly these PATR clusters represent expressed transcripts, we calculated pseudo-bulk TPM across the 3 major cell types. Among 7,597 genes with TPM ≥1, 5,864 genes (∼77%) exhibited at least 2 UMIs, indicating that despite the low proportion of PATRs s in scRNA-seq data, their coverage encompasses the majority of expressed genes. Of the 620 transcripts (517 genes) with novel transcription end sites in *Drosophila* larvae (Supplementary Fig. 7 bottom left), 446 transcripts (372 genes) were confirmed at the single-cell level by the PATR clusters, and 4.2% to 4.6% of the UMIs were associated with these newly discovered transcription end sites across the 3 major cell types (Fig. 6c). Of the 372 genes supported by the PATR clusters and containing at least 1 novel transcription end site, the 233 genes—representing more than half—used novel transcription end sites as their dominant polyadenylation site (Fig. 6d, left). For 116 of these genes, the novel transcription end sites remained dominant across all cell types, indicating transcripts that may be missing from the current reference annotation, as exemplified in the *RpS27* locus (Fig. 6d and e). In other cases, newly identified transcription end sites were significantly enriched in specific cell types—for example, in the *AnxB9* locus, the novel transcription end site was particularly abundant in PH (Fig. 6f).

Overall, using PATRs s from scRNA-seq data, we mapped poly(A) sites at or near the 3′ ends of both reference and novel transcripts in each hemocyte type. Certain genes relied heavily on these novel transcription end sites—either universally or in specific subpopulations—indicating unannotated transcripts in the reference annotation and cell type–specific APA in *Drosophila* larvae.

## Discussion

In this study, we combined ONT long-read cDNA sequencing and Illumina short-read RNA-seq to obtain a high-resolution view of *Drosophila* larval transcriptomes under normal and parasitic wasp–infested conditions. By exploiting the advantages of both platforms, we generated a hybrid transcriptome that revealed numerous novel lncRNAs, alternative transcription start and end sites, and provided refined gene annotations. The large number of novel isoforms within existing gene loci and transcripts (679 transcripts with alternative 5′ or 3′ ends and 93 new isoforms within existing gene loci) and 408 new transcripts from novel gene loci likely reflects the use of long-read sequencing, which enables direct reconstruction of full-length transcript structures—including alternative splice junctions, exon usage, and transcript boundaries—that are difficult to resolve using short-read RNA-seq alone. In contrast, many existing annotations are largely derived from short-read datasets, and we anticipate that the integration of long-read datasets could further improve reference transcriptome repertoire. Our updated gene model significantly improved the analysis of DEGs, revealed lineage-specific lncRNA markers in LMs, and highlighted newly discovered APA events validated at single-cell resolution.

A key strength of our approach lies in the high-confidence detection of full-length transcripts and isoforms, facilitated by long-read sequencing. The identification of both 5′ and 3′ boundaries with improved accuracy (Fig. 1d to f; Supplementary Fig. 3) underscores the importance of using ONT data to annotate promoter regions, UTRs, and APA sites. Furthermore, the systematic correction of sequencing errors and potential splice site misalignments using short-read dataset and the detection and removal of ONT-specific intra-priming artifacts ensured the fidelity of our final transcript models (Fig. 2b). Together, these improvements allowed for more accurate interpretation of gene expression and revealed new insights into *Drosophila* larval hematopoiesis and immune responses. Our results also highlight the critical importance of validating newly identified isoforms and RNAs, as failure to do so can perpetuate errors in downstream analyses.

Despite the clear advantages of the single-cell strategy, single-cell RNA-seq technologies still capture only partial transcripts—mainly the 3′ ends—making them susceptible to missing or underestimating certain isoforms. Although we used PATRs s to validate novel transcription end sites (Fig. 6), the low proportion of these reads may limit robust quantification. In the future, single-cell long-read sequencing platforms may overcome many of these challenges, providing direct access to the full-length transcriptome per cell and enabling in-depth studies of splicing regulation in *Drosophila* larvae.

Looking ahead, our expanded transcriptome annotations and identification of lncRNAs hold great promise for unraveling noncoding regulatory mechanisms in *Drosophila*. We found that numerous lncRNAs are highly induced at later stages of infestation and are specifically expressed along the LM lineage by trajectory analyses (Figs. 4e and 5c). RNAi-mediated knockdown of lncRNA:CR44316 or lncRNA:CR44948 further demonstrated their essential role in LM differentiation (Fig. 5e). However, in-depth functional studies—such as CRISPR-based deletions, rescue experiments, and advanced epigenetic or transcriptomic assays—are needed to define the precise molecular pathways that lncRNAs modulate during hematopoiesis and immune activation.

In conclusion, our hybrid transcriptome assembly greatly refines gene and isoform annotations in *Drosophila* larvae and reveals a wealth of novel lncRNAs involved in hemocyte differentiation and immune responses. These results underscore the importance of 5′ and 3′ boundaries mapping for both coding and noncoding gene models, demonstrate the power of hybrid sequencing to improve transcriptome analyses, and highlight lncRNAs as key regulatory components of *Drosophila* immunity. Finally, our approach may also serve as a blueprint for refining gene annotations and discovering underappreciated regulatory RNAs in other species beyond *Drosophila*.

## Supplementary Material

iyag208_Supplementary_Data

## Data Availability

The data that we generated in this project have been deposited in the National Institutes of Health, NCBI BioProject database (accession number: PRJNA1301980) and Gene Expression Omnibus (accession number: GSE329936), and the Korean BioData Station (K-BDS, https://kbds.re.kr/) under the accession number KAP242477. In-house R and Python codes that were implemented in this study are available on GitHub (https://github.com/Kyung-TaeLee/Dmel_hybrid_transcriptome_singlecell). Supplemental material available at GENETICS online.
