# Supplementary material for: A hybrid transcriptome assembly reveals coding and noncoding landscapes in Drosophila hemocytes
Source: Genetics. 2026 Aug 11;234(2):iyag208. doi: 10.1093/genetics/iyag208 (PMC13643772; doi:10.1093/genetics/iyag208)
Supplement: Supplementary file 1 [file iyag208_supplementary_data.zip › Supplemental_Figure_Legends_GENETICS-2026-309442.docx]

**Supplementary Figure Legends**

**Supplementary Figure 1. Summary of sequencing statistics**

**(A)** N50 of ONT reads from each condition. **(B-C)** Number of reads **(B)** and bases **(C)** mapped to genome of *Drosophila* melanogaster for each sequencing platform across conditions. WT, wild-type; wIF, wasp Infestation.

**Supplementary Figure 2. Schematic illustration of hybrid transcriptome assembly in *Drosophila* larvae**

Schematic illustration of hybrid transcriptome assembly pipeline integrating ONT and Illumina sequencing data. The Illumina RNA-seq workflow includes pre-processing, genome alignment, de novo assembly and transcriptome merging (left). The ONT workflow involves pre-processing, read re-orientation, error correction using 21-mers from Illumina sequencing, de novo assembly with exon-junction update based on information from Illumina sequencing and transcriptome merging (right). Transcriptomes from each sequencing platform were then compared and classified as “Tier 1” if they exhibited exonic overlap (for single-exon transcripts) or shared same intron chain (for multi-exon transcripts); otherwise, they were classified as “Tier 2”. ONT “Tier 1” and “Tier 2” transcriptomes, along with Illumina “Tier 2” transcriptomes, were merged to define the initial hybrid transcriptome. Trx, transcripts. Created with bioRender (https://www.biorender.com).

**Supplementary Figure 3. Enrichment of known promoter motifs near the transcription start sites**

The frequencies of downstream promoter elements (DPE, consensus sequence: RGWYV) and TATA box (consensus sequence: TATAAA) near the transcription start sites of transcripts, as shown in Figure 1D.

**Supplementary Figure 4. Reproducibility of gene and isoform expression between biological replicates and sequencing platforms**

**(A)** Principal component analysis was performed using log_2_ transformed gene (left) and isoform (right) expressions (counts per million mapped reads, CPM) measured by ONT data. The plot displays principal component (PC) 1 and 2 along x- and y-axes, respectively, with percent of variance explained by each principal component indicated in parentheses. **(B)** Gene (top) and isoform expression levels (bottom) measured by Illumina (log_2_ transformed transcripts per million mapped reads) and ONT (log_2_ transformed CPM) data are projected onto x- and y-axes, respectively. Pearson correlation coefficients (*R*) of expression between the two sequencing platforms are indicated for each condition.

**Supplementary Figure 5. Expression levels of known tissue and cell type specific markers in short and long-read RNA-seq data**

**(A-B)** Heatmaps depict scaled expression of previously reported lineage **(A)** and cell type specific **(B)** marker genes in ONT **(left)** and Illumina **(right)** sequencing data**.** WT, wild-type; wIF, wasp infestation.

**Supplementary Figure 6. Heuristic thresholding to identify ONT specific intra-priming events based on CDS disruption events by novel transcript ends**

Transcripts with putative transcription start or end sites that disrupt annotated CDS were analyzed to establish heuristic thresholds for identifying ONTS specific intra-priming events. **(A)** To identify potential 5’ intra-priming by strand switching primer, local alignment scores of the transcription start sites flanking sequences ($\pm$ 25 nt around putative transcription start sites disrupting annotated CDS, *n* =30) to strand switching primer sequence were compared to those of references transcripts. A heuristic threshold of 25 was established based on manual observation. **(B)** For 3’ intra-priming by VN primer with olilgo(dT), base compositions near transcription end sites (1nt upstream to 19 nt downstream of the putative transcription end sites disrupting annotated CDS, *n*= 66) were analyzed. A heuristic threshold of an adenosine frequency 0.8 was set based on manual observation.

**Supplementary Figure 7. Identification of novel transcripts through hybrid transcriptome assembly approach**

Overview of the pipeline to identify and classify novel transcripts in *Drosophila* larvae. Starting with the filtered transcriptome from the final steps outlined in Figure 2A (*n*= 18,801), the transcriptome was initially compared against the BDGP reference gene model using GFFcompare. (YES – to the left) A total of 12,513 transcript exhibiting an exact match of intron chain with reference transcripts (for single-exon transcripts, more than 80% exonic overlap) were further filtered using more stringent expression thresholds (ONT CPM and Illumina TPM ≥ 3 for lncRNAs; otherwise, threshold was set as 5). The schematic below illustrates the strategy for classifying transcripts with novel transcription start or end sites, or both, applying thresholds of greater than 100 nt and 15 nt for distances from the closest reference 5’ and 3’ ends, respectively. The statistics for transcripts with novel boundaries (5’ or 3’ ends) are presented below. (NO – to the right) Transcripts that do not fully match but partially overlap with known lncRNA loci (Box titled “Exonic overlap with reference lncRNA”) or do not overlap any reference transcripts on the same strand (Box titled “Novel loci RNA”) were evaluated of protein-coding potential using *in silico* methods (coding potential calculator, CPC, and coding potential assessment tool, CPAT), leading to identification of novel isoforms from reference (*Ref.*) lncRNA loci and novel transcripts from novel loci. The schematics illustrating the strategy of transcript classification in the gray boxes were adapted from GFFcompare manual. Trx, transcripts.

**Supplementary Figure 8. Potential bi-functional genes in *Drosophila* larvae**

**(A-F)** Schematics of genes with novel transcription start sites that disrupt annotated CDS (Ref_TSS), potentially resulting in non-coding isoform and functioning as bi-functional genes in *Drosophila* larvae. Read coverages from both sequencing platforms across all examined conditions are also depicted for these genes, including *CG3552* **(A)** and *lawc* **(B)** highlighted in Figure 2D. WT, wild-type; wIF, wasp infestation. **(F)** *In vivo* validation of novel transcription start sites (TSS). Expression levels were normalized with rp49.

**Supplementary Figure 9. Non-coding nature of newly identified lncRNAs and differentially expressed lncRNAs at later stage of wasp-infestation in circulating hemocytes**

**(A)** Tau specificity index of gene expression for reference protein-coding genes, reference lncRNA genes, and novel lncRNA genes in Illumina and ONT data. **(B)** Schematic illustration of genic (left) and intergenic (right) lncRNA subtypes that are classified based on their neighboring protein-coding genes (PCG), along with their composition in reference (*Ref.*) and novel lncRNA genes. **(C)** Expression and log2 fold change ($\frac{Infested expression}{Normal expression}$) of lncRNA genes in circulating hemocytes of 120 h after egg laying are shown. Three representative lncRNAs that are strongly induced in wasp infested condition (Figure 3) are highlighted.

**Supplementary Figure 10. scRNA-seq analysis of *Drosophila* larval hemocytes under normal and wasp infested conditions**

**(A)** Number of cells, unique molecular identifiers (UMIs), genes, and mitochondrial (MT) contents for each sequencing library are shown, with colors indicating the sample of origin. **(B)** t-SNE plot showing two-dimensional projection of cells categorized by infestation status, as in Figure 4D. **(C)** (Top) Number and (bottom) relative proportion of cell types in hemocytes from normal (left) and infested (right) conditions. WT, wild-type; wIF, wasp infestation.

**Supplementary Figure 11. Expression pattern of cell-type specific protein-coding markers in *Drosophila* larval hemocytes**

The expression levels of known protein-coding markers in *Drosophila* larvae hemocytes in two-dimensional t-SNE plots. Colors indicate scaled gene expression. Prohemocyte markers: *Anc*e, *IM18*, and *Tep4*; Plasmatocyte markers: *Hml*, *Pxn*, and *NimC1*; Lamellocyte markers: *msn* and *atilla*; Crystal cell markers: *PPO1* and *PPO2*; Posterior signaling center markers: *Antp*.

**Supplementary Figure 12. Expression level of cell-cycle markers in circulating hemocytes**

**(A)** Expression level of three cell-cycle markers (*stg*, *polo*, and *scra*) in each subcluster of circulating hemocytes. **(B)** A three-dimensional UMAP projection of circulating hemocytes grouped by infestation status, as in Figure 5A and B. WT, wild-type; wIF, wasp infestation.

**Supplementary Figure 13. *In-vivo* validation of lncRNAs expression.**

**(A)** lncRNA expression was validated by SABER-FISH method in the circulating hemocyte. *CR44948* (top; magenta), *CR44316* (middle; magenta), or *novel-57* (bottom; magenta) expression was mostly detected in lamellocytes with *atilla* RNA expression (green). Quantification of each SABER-FISH probes intensity on the right side. Mann-Witney test was used for analysis. ***, *p<0.001*. ****, *p<0.0001*. **(B)** Confirmation of RNAi efficiency using the pan-hemocyte driver (*Srp-Gal4, HmlΔ-Gal4; UAS-CR44948 RNAi* or *UAS-CR44316 RNAi*).

**Supplementary Figure 14. Distribution of length of soft-clipped regions and transcript 3’ends support by poly(A) tailed reads in single-cell RNA-seq data**

**(A)** Distribution of the length of soft-clipped regions in Drop-seq data across three major cell types (LM, PH, and PM). **(B)** Distribution of the number of transcripts 3’ends supported by unique poly(A) tailed reads. The minimum and maximum number of unique molecular identifiers assigned to transcripts 3’ends (bottom) and total number of genes and transcripts supported by at least 2 unique molecular identifiers are indicated in the plot (top). Trx, transcripts.

**Supplementary Table legends**

**Supplementary Table 1. Statistics of sequencing reads**

The number of total reads, bases, filtered reads, mapped reads, and bases are summarized for each library of ONT **(A)** and Illumina sequencing data **(B)** generated or utilized in this study. GEO accession numbers and indications of whether the data were generated in this study are also provided.

**Supplementary Table 2. RT-qPCR primer sequences**

RT-qPCR primer sequences utilized to detect RNA expression level of lncRNAs and protein-coding gene in circulating hemocyte under normal (120h after egg laying) and wasp infested condition (48 h post infestation), as shown in Figure 3C, bottom.

**Supplementary Table 3. Drop-seq cell counts**

Number of cells selected for downstream analysis in each sequencing library of Drop-seq data.

**Supplementary Table 4. SABER-FISH probe sequences**

Probe sequences to detect in-situ RNA-expression of lncRNAs through SABER-FISH in circulating hemocytes under normal (120h after egg laying) and wasp infested condition (48 h post infestation), as shown in Supplementary Figure 13.

**Supplementary Table 5. RNAi sequences**

RNAi sequences used to knock down lncRNA expression in plasmatocytes, as shown in Figure 5E, are listed.
