# Supplementary material for: A hybrid transcriptome assembly reveals coding and noncoding landscapes in Drosophila hemocytes
Source: Genetics. 2026 Aug 11;234(2):iyag208. doi: 10.1093/genetics/iyag208 (PMC13643772; doi:10.1093/genetics/iyag208)
Supplement: Supplementary file 1 [file iyag208_supplementary_data.zip › Supplementary_Figure_10_GENETICS-2026-309442.pdf]

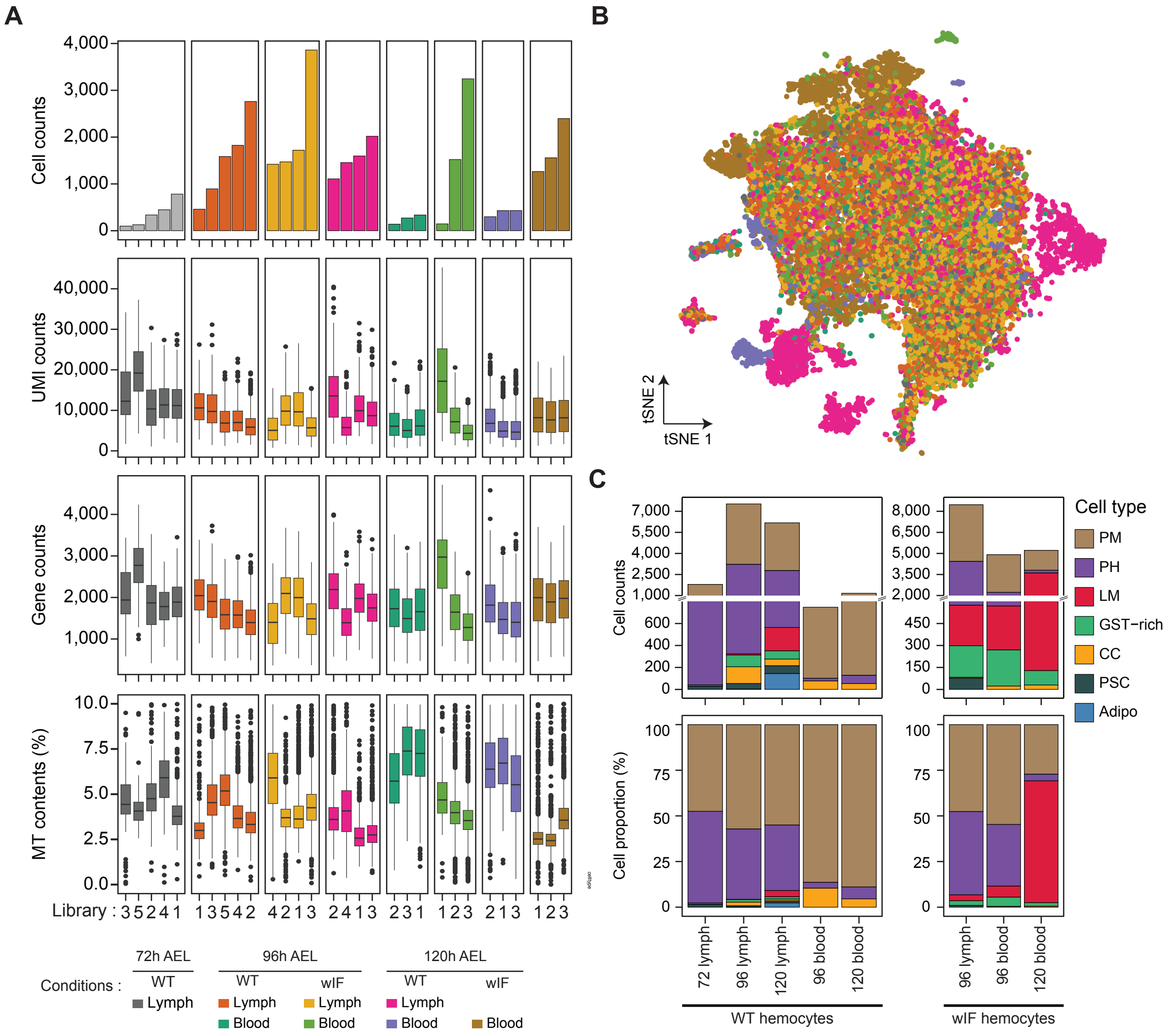
A
B
C
Cell counts
UMI counts
Gene counts
MT contents (%)
Library : 3 5 2 4 1 1 3 5 4 2 4 2 1 3 2 4 1 3 2 3 1 1 2 3 2 1 3 1 2 3
Conditions :
72h AEL
96h AEL
120h AEL
WT
WT
wIF
WT
wIF
Lymph
Lymph
Lymph
Lymph
Blood
Blood
Blood
Blood
tSNE 2
tSNE 1
Cell counts
Cell proportion (%)
Cell type
PM
PH
LM
GST−rich
CC
PSC
Adipo
72 lymph
96 lymph
120 lymph
96 blood
120 blood
96 lymph
96 blood
120 blood
WT hemocytes
wIF hemocytes
