# Supplementary material for: A hybrid transcriptome assembly reveals coding and noncoding landscapes in Drosophila hemocytes
Source: Genetics. 2026 Aug 11;234(2):iyag208. doi: 10.1093/genetics/iyag208 (PMC13643772; doi:10.1093/genetics/iyag208)
Supplement: Supplementary file 1 [file iyag208_supplementary_data.zip › Supplementary_Figure_11_GENETICS-2026-309442.pdf]

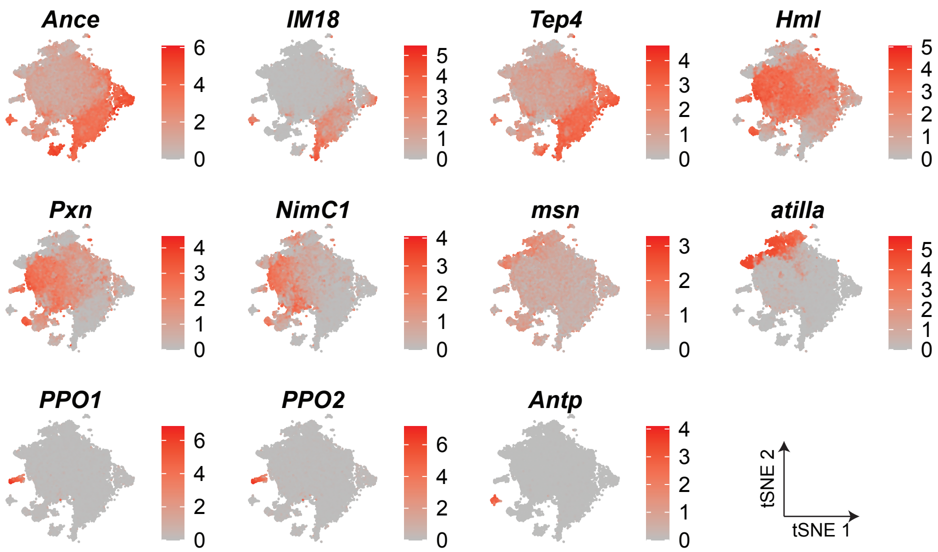
Ance
6
4
2
0
IM18
5
4
3
2
1
0
Tep4
4
3
2
1
0
Hml
5
4
3
2
1
0
Pxn
4
3
2
1
0
NimC1
4
3
2
1
0
msn
3
2
1
0
atilla
5
4
3
2
1
0
PPO1
6
4
2
0
PPO2
6
4
2
0
Antp
4
3
2
1
0
tSNE 2
tSNE 1
