# Supplementary material for: A hybrid transcriptome assembly reveals coding and noncoding landscapes in Drosophila hemocytes
Source: Genetics. 2026 Aug 11;234(2):iyag208. doi: 10.1093/genetics/iyag208 (PMC13643772; doi:10.1093/genetics/iyag208)
Supplement: Supplementary file 1 [file iyag208_supplementary_data.zip › Supplementary_Figure_12_GENETICS-2026-309442.pdf]

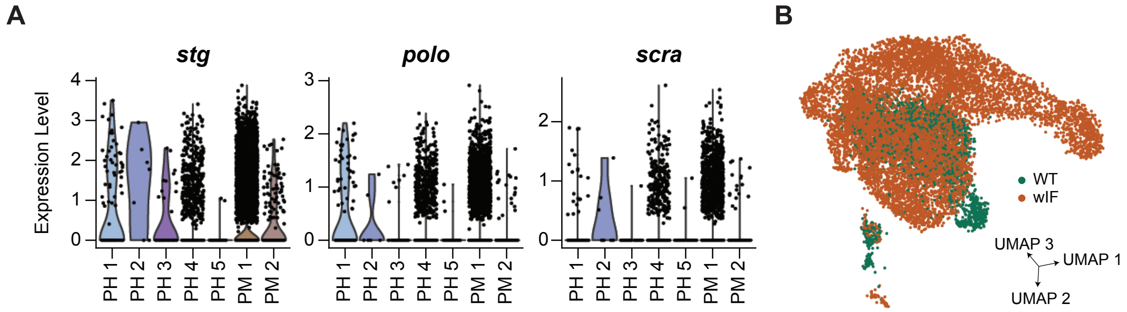
A
stg
polo
scra
Expression Level
PH 1
PH 2
PH 3
PH 4
PH 5
PM 1
PM 2
B
WT
wIF
UMAP 3
UMAP 1
UMAP 2
