# Supplementary material for: A hybrid transcriptome assembly reveals coding and noncoding landscapes in Drosophila hemocytes
Source: Genetics. 2026 Aug 11;234(2):iyag208. doi: 10.1093/genetics/iyag208 (PMC13643772; doi:10.1093/genetics/iyag208)
Supplement: Supplementary file 1 [file iyag208_supplementary_data.zip › Supplementary_Figure_13_GENETICS-2026-309442.pdf]

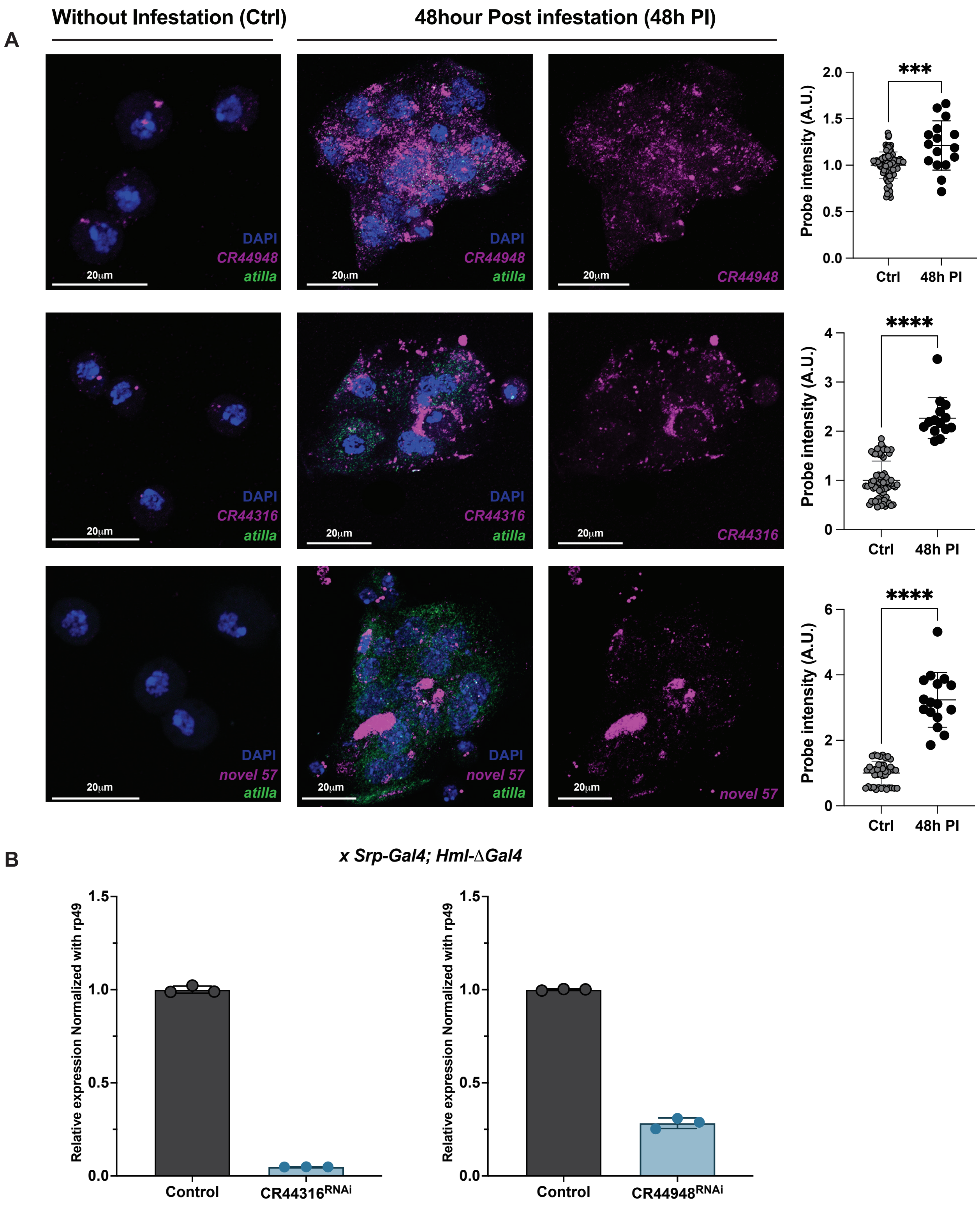

A
Without Infestation (Ctrl)
48hour Post infestation (48h PI)
DAPI
CR44948
atilla
20μm
CR44948
Probe intensity (A.U.)
***
Ctrl
48h PI
DAPI
CR44316
atilla
CR44316
****
DAPI
novel 57
atilla
novel 57
****
B
x Srp-Gal4; Hml-ΔGal4
Relative expression Normalized with rp49
Control
CR44316RNAi
CR44948RNAi
