# Supplementary material for: A hybrid transcriptome assembly reveals coding and noncoding landscapes in Drosophila hemocytes
Source: Genetics. 2026 Aug 11;234(2):iyag208. doi: 10.1093/genetics/iyag208 (PMC13643772; doi:10.1093/genetics/iyag208)
Supplement: Supplementary file 1 [file iyag208_supplementary_data.zip › Supplementary_Figure_14_GENETICS-2026-309442.pdf]

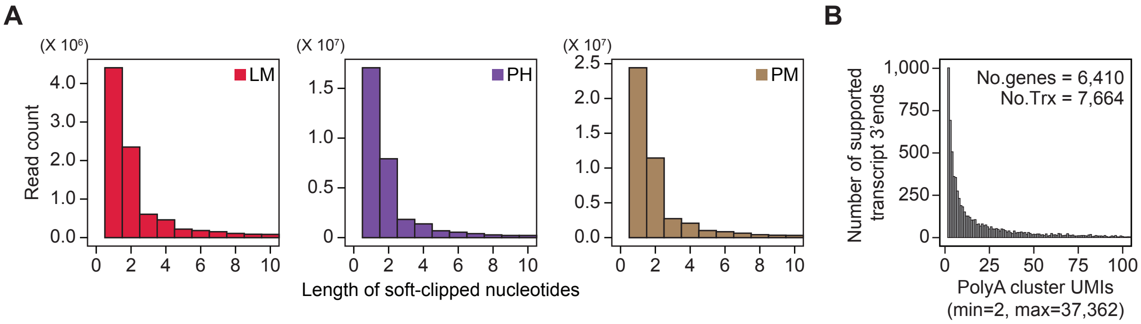
A
(X 10⁶)
LM
Read count
4.0
3.0
2.0
1.0
0.0
0 2 4 6 8 10
(X 10⁷)
PH
1.5
1.0
0.5
0
0 2 4 6 8 10
Length of soft-clipped nucleotides
(X 10⁷)
PM
2.5
2.0
1.5
1.0
0.5
0.0
0 2 4 6 8 10
B
Number of supported transcript 3'ends
No.genes = 6,410
No.Trx = 7,664
1,000
750
500
250
0
0 25 50 75 100
PolyA cluster UMIs
(min=2, max=37,362)
