# Supplementary material for: A hybrid transcriptome assembly reveals coding and noncoding landscapes in Drosophila hemocytes
Source: Genetics. 2026 Aug 11;234(2):iyag208. doi: 10.1093/genetics/iyag208 (PMC13643772; doi:10.1093/genetics/iyag208)
Supplement: Supplementary file 1 [file iyag208_supplementary_data.zip › Supplementary_Figure_1_GENETICS-2026-309442.pdf]

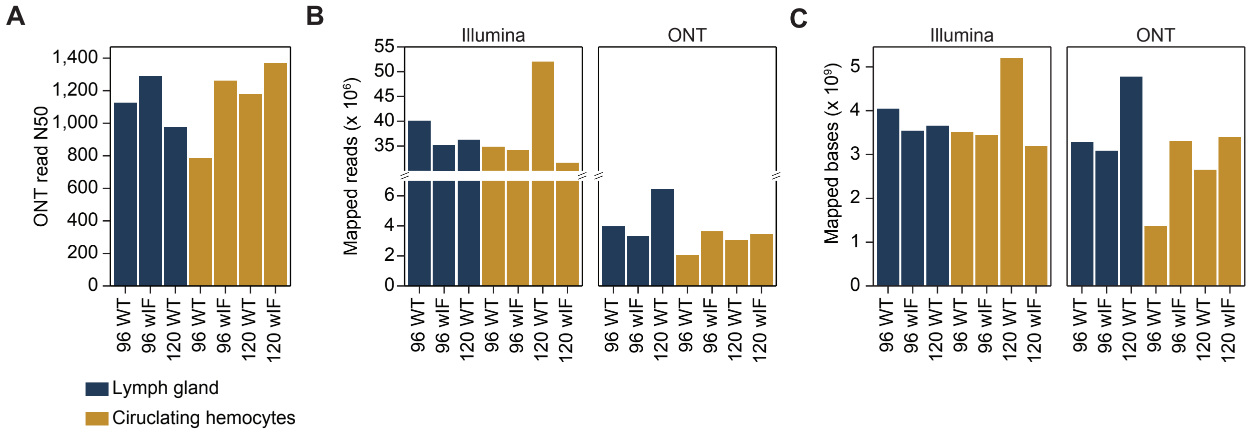
A
ONT read N50
1,400
1,200
1,000
800
600
400
200
0
96 WT
96 wIF
120 WT
96 WT
96 wIF
120 WT
120 wIF
Lymph gland
Ciruclating hemocytes
B
Illumina
ONT
Mapped reads (x 10⁶)
55
50
45
40
35
6
4
2
0
C
Illumina
ONT
Mapped bases (x 10⁹)
5
4
3
2
1
0
