# Supplementary material for: A hybrid transcriptome assembly reveals coding and noncoding landscapes in Drosophila hemocytes
Source: Genetics. 2026 Aug 11;234(2):iyag208. doi: 10.1093/genetics/iyag208 (PMC13643772; doi:10.1093/genetics/iyag208)
Supplement: Supplementary file 1 [file iyag208_supplementary_data.zip › Supplementary_Figure_2_GENETICS-2026-309442.pdf]

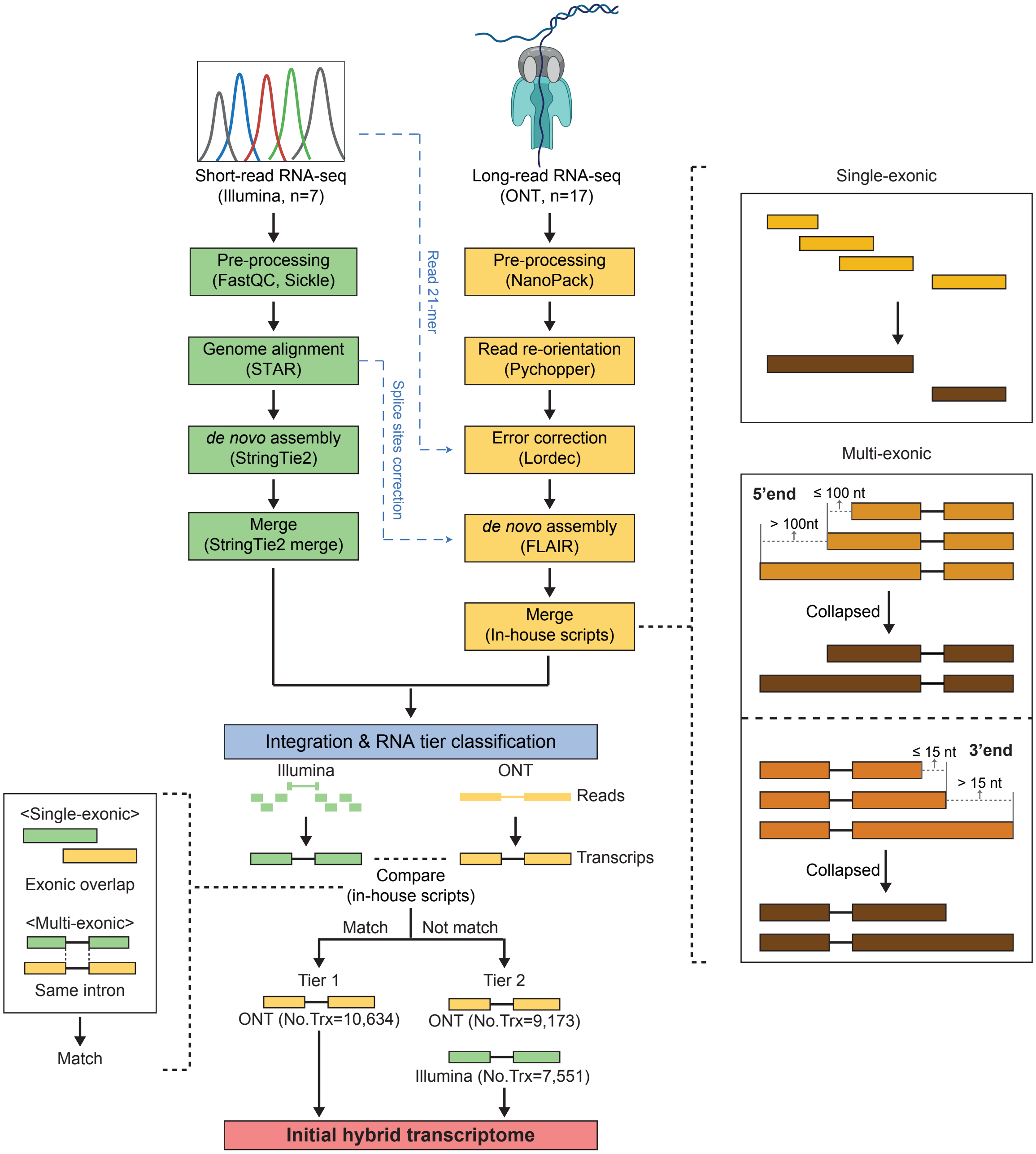
Short-read RNA-seq
(Illumina, n=7)
Long-read RNA-seq
(ONT, n=17)
Read 21-mer
Pre-processing
(FastQC, Sickle)
Genome alignment
(STAR)
de novo assembly
(StringTie2)
Merge
(StringTie2 merge)
Splice sites correction
Pre-processing
(NanoPack)
Read re-orientation
(Pychopper)
Error correction
(Lordec)
de novo assembly
(FLAIR)
Merge
(In-house scripts)
Single-exonic
Multi-exonic
5'end
≤ 100 nt
> 100nt
Collapsed
≤ 15 nt
3'end
> 15 nt
Collapsed
Integration & RNA tier classification
Illumina
ONT
Reads
Transcrips
Compare
(in-house scripts)
<Single-exonic>
Exonic overlap
<Multi-exonic>
Same intron
Match
Match
Not match
Tier 1
ONT (No.Trx=10,634)
Tier 2
ONT (No.Trx=9,173)
Illumina (No.Trx=7,551)
Initial hybrid transcriptome
