# Supplementary material for: A hybrid transcriptome assembly reveals coding and noncoding landscapes in Drosophila hemocytes
Source: Genetics. 2026 Aug 11;234(2):iyag208. doi: 10.1093/genetics/iyag208 (PMC13643772; doi:10.1093/genetics/iyag208)
Supplement: Supplementary file 1 [file iyag208_supplementary_data.zip › Supplementary_Figure_3_GENETICS-2026-309442.pdf]

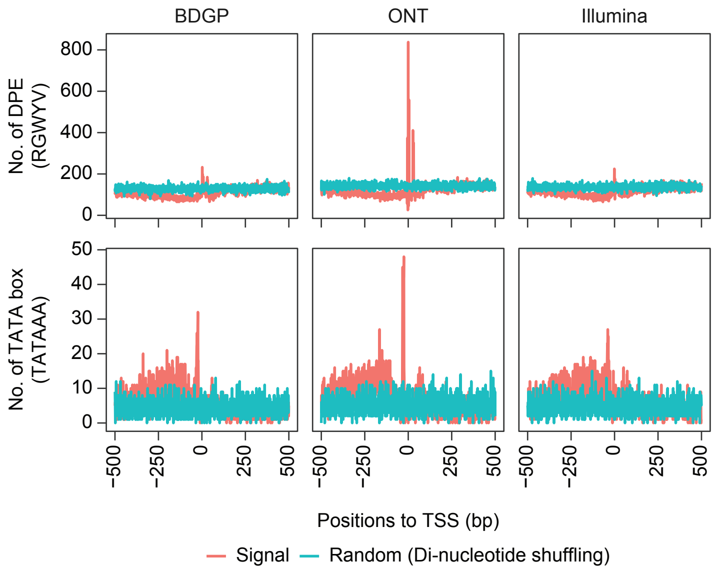

BDGP
ONT
Illumina
No. of DPE (RGWYV)
0
200
400
600
800
No. of TATA box (TATAAA)
0
10
20
30
40
50
−500
−250
0
250
500
Positions to TSS (bp)
Signal
Random (Di-nucleotide shuffling)
