# Supplementary material for: A hybrid transcriptome assembly reveals coding and noncoding landscapes in Drosophila hemocytes
Source: Genetics. 2026 Aug 11;234(2):iyag208. doi: 10.1093/genetics/iyag208 (PMC13643772; doi:10.1093/genetics/iyag208)
Supplement: Supplementary file 1 [file iyag208_supplementary_data.zip › Supplementary_Figure_4_GENETICS-2026-309442.pdf]

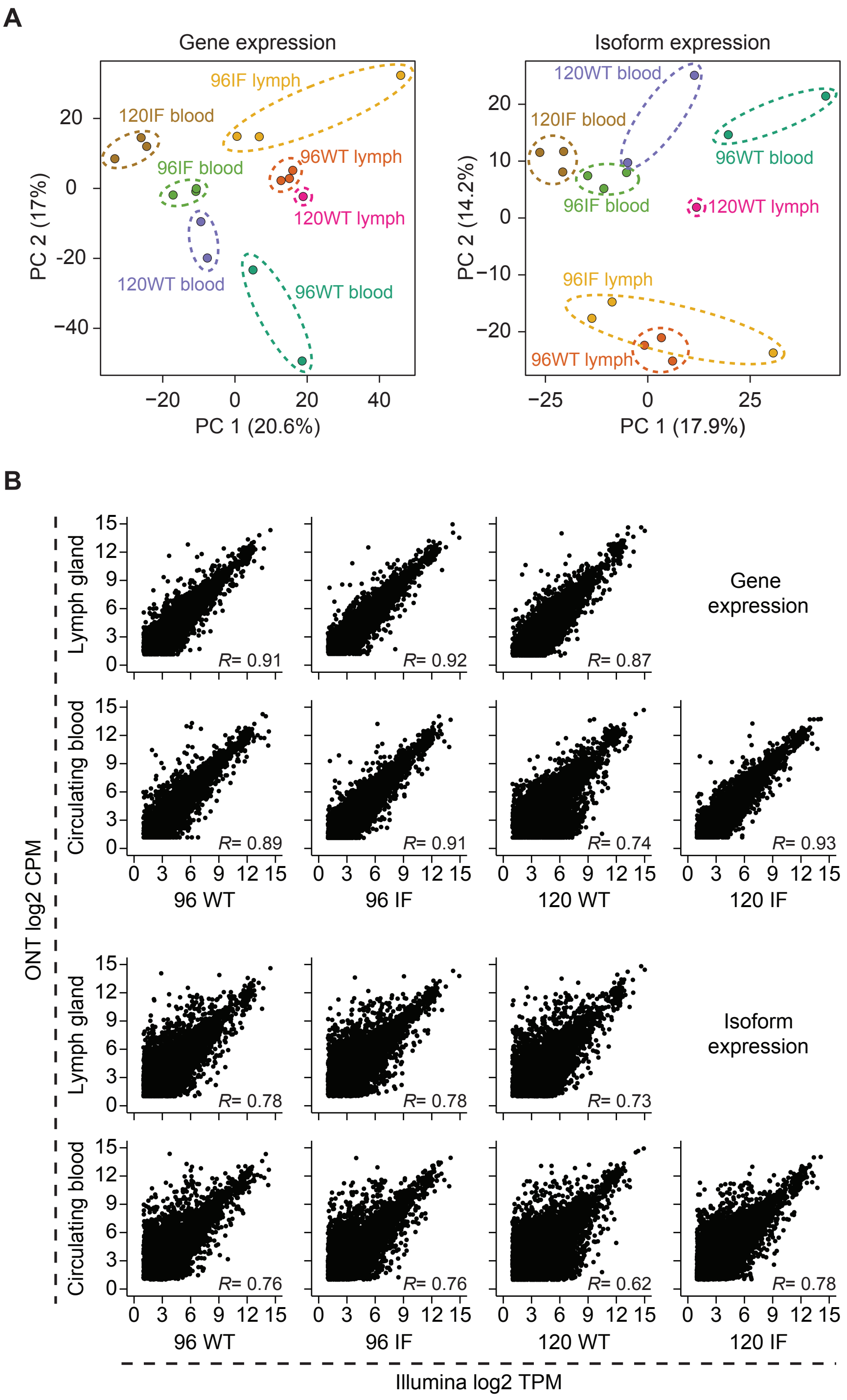
A
Gene expression
96IF lymph
120IF blood
96WT lymph
96IF blood
120WT lymph
120WT blood
96WT blood
PC 2 (17%)
PC 1 (20.6%)
Isoform expression
120WT blood
120IF blood
96WT blood
96IF blood
120WT lymph
96IF lymph
96WT lymph
PC 2 (14.2%)
PC 1 (17.9%)
B
Lymph gland
Circulating blood
Gene expression
Isoform expression
R= 0.91
R= 0.92
R= 0.87
R= 0.89
R= 0.91
R= 0.74
R= 0.93
R= 0.78
R= 0.78
R= 0.73
R= 0.76
R= 0.76
R= 0.62
R= 0.78
96 WT
96 IF
120 WT
120 IF
ONT log2 CPM
Illumina log2 TPM
