# Supplementary material for: A hybrid transcriptome assembly reveals coding and noncoding landscapes in Drosophila hemocytes
Source: Genetics. 2026 Aug 11;234(2):iyag208. doi: 10.1093/genetics/iyag208 (PMC13643772; doi:10.1093/genetics/iyag208)
Supplement: Supplementary file 1 [file iyag208_supplementary_data.zip › Supplementary_Figure_5_GENETICS-2026-309442.pdf]

A

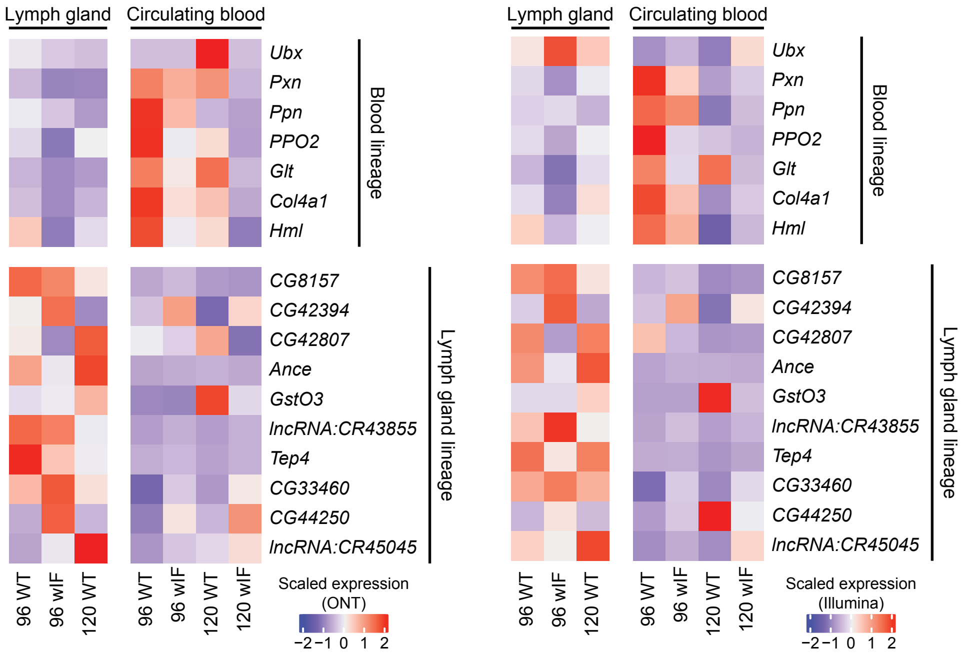

Lymph gland
Circulating blood
Ubx
Pxn
Ppn
PPO2
Glt
Col4a1
Hml
Blood lineage
CG8157
CG42394
CG42807
Ance
GstO3
lncRNA:CR43855
Tep4
CG33460
CG44250
lncRNA:CR45045
Lymph gland lineage
96 WT
96 wIF
120 WT
120 wIF
Scaled expression (ONT)
−2 −1 0 1 2
Scaled expression (Illumina)

B

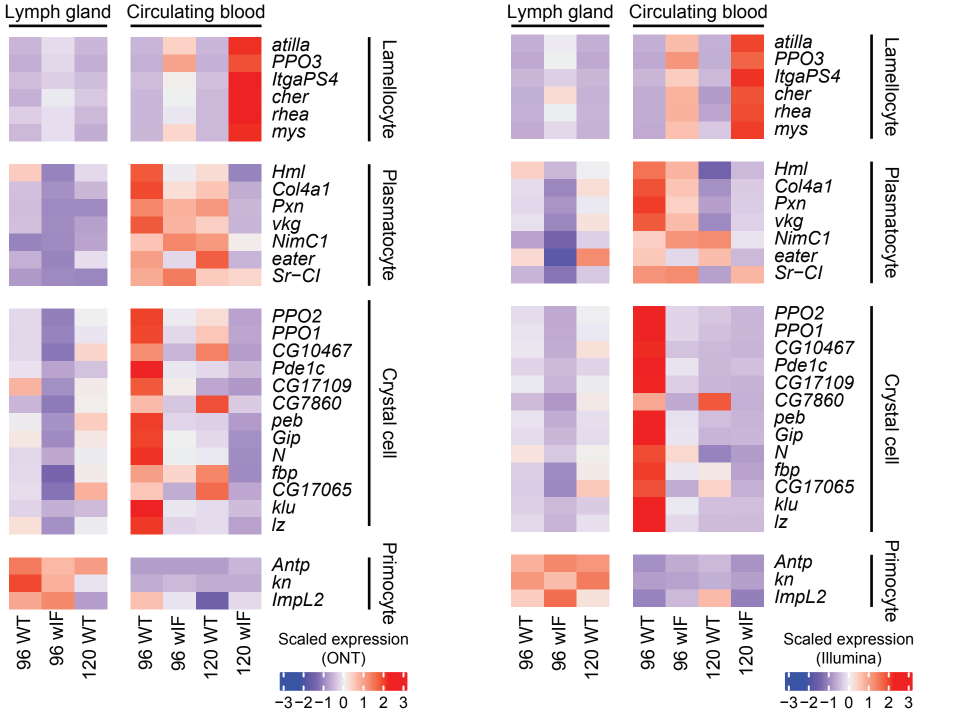

Lymph gland
Circulating blood
atilla
PPO3
ItgaPS4
cher
rhea
mys
Lamellocyte
Hml
Col4a1
Pxn
vkg
NimC1
eater
Sr−CI
Plasmatocyte
PPO2
PPO1
CG10467
Pde1c
CG17109
CG7860
peb
Gip
N
fbp
CG17065
klu
lz
Crystal cell
Antp
kn
ImpL2
Primocyte
96 WT
96 wIF
120 WT
120 wIF
Scaled expression (ONT)
−3 −2 −1 0 1 2 3
Scaled expression (Illumina)
