# Supplementary material for: A hybrid transcriptome assembly reveals coding and noncoding landscapes in Drosophila hemocytes
Source: Genetics. 2026 Aug 11;234(2):iyag208. doi: 10.1093/genetics/iyag208 (PMC13643772; doi:10.1093/genetics/iyag208)
Supplement: Supplementary file 1 [file iyag208_supplementary_data.zip › Supplementary_Figure_6_GENETICS-2026-309442.pdf]

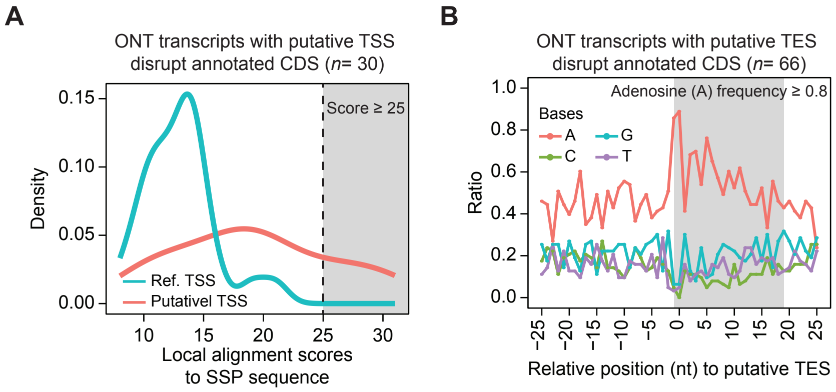
A
ONT transcripts with putative TSS
disrupt annotated CDS (n= 30)
Density
0.15
0.10
0.05
0.00
Score ≥ 25
Ref. TSS
Putativel TSS
10
15
20
25
30
Local alignment scores
to SSP sequence
B
ONT transcripts with putative TES
disrupt annotated CDS (n= 66)
Adenosine (A) frequency ≥ 0.8
Bases
A
G
C
T
Ratio
1.0
0.8
0.6
0.4
0.2
0.0
−25
−20
−15
−10
−5
0
5
10
15
20
25
Relative position (nt) to putative TES

Bases A T C G
