# Supplementary material for: A hybrid transcriptome assembly reveals coding and noncoding landscapes in Drosophila hemocytes
Source: Genetics. 2026 Aug 11;234(2):iyag208. doi: 10.1093/genetics/iyag208 (PMC13643772; doi:10.1093/genetics/iyag208)
Supplement: Supplementary file 1 [file iyag208_supplementary_data.zip › Supplementary_Figure_7_GENETICS-2026-309442.pdf]

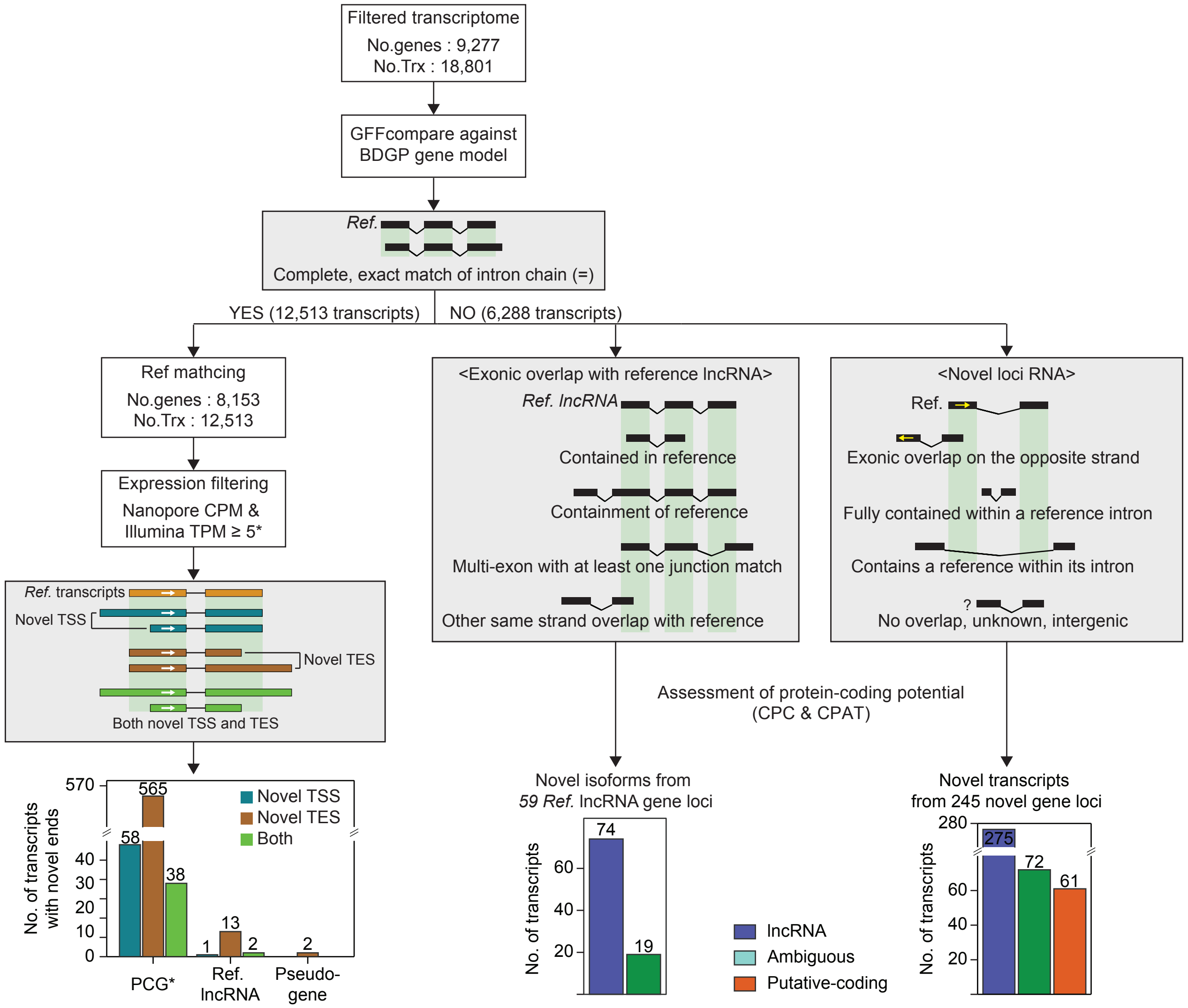

Filtered transcriptome
No.genes : 9,277
No.Trx : 18,801
GFFcompare against
BDGP gene model
Ref.
Complete, exact match of intron chain (=)
YES (12,513 transcripts)
NO (6,288 transcripts)
Ref mathcing
No.genes : 8,153
No.Trx : 12,513
Expression filtering
Nanopore CPM &
Illumina TPM ≥ 5*
Ref. transcripts
Novel TSS
Novel TES
Both novel TSS and TES
<Exonic overlap with reference lncRNA>
Ref. lncRNA
Contained in reference
Containment of reference
Multi-exon with at least one junction match
Other same strand overlap with reference
<Novel loci RNA>
Ref.
Exonic overlap on the opposite strand
Fully contained within a reference intron
Contains a reference within its intron
?
No overlap, unknown, intergenic
Assessment of protein-coding potential
(CPC & CPAT)
No. of transcripts
with novel ends
570
565
58
38
40
30
20
10
0
1
13
2
2
Novel TSS
Novel TES
Both
PCG*
Ref.
lncRNA
Pseudo-
gene
Novel isoforms from
59 Ref. lncRNA gene loci
No. of transcripts
74
19
60
40
20
lncRNA
Ambiguous
Putative-coding
Novel transcripts
from 245 novel gene loci
No. of transcripts
280
275
72
61
60
40
20
