# Supplementary material for: A hybrid transcriptome assembly reveals coding and noncoding landscapes in Drosophila hemocytes
Source: Genetics. 2026 Aug 11;234(2):iyag208. doi: 10.1093/genetics/iyag208 (PMC13643772; doi:10.1093/genetics/iyag208)
Supplement: Supplementary file 1 [file iyag208_supplementary_data.zip › Supplementary_Figure_8_GENETICS-2026-309442.pdf]

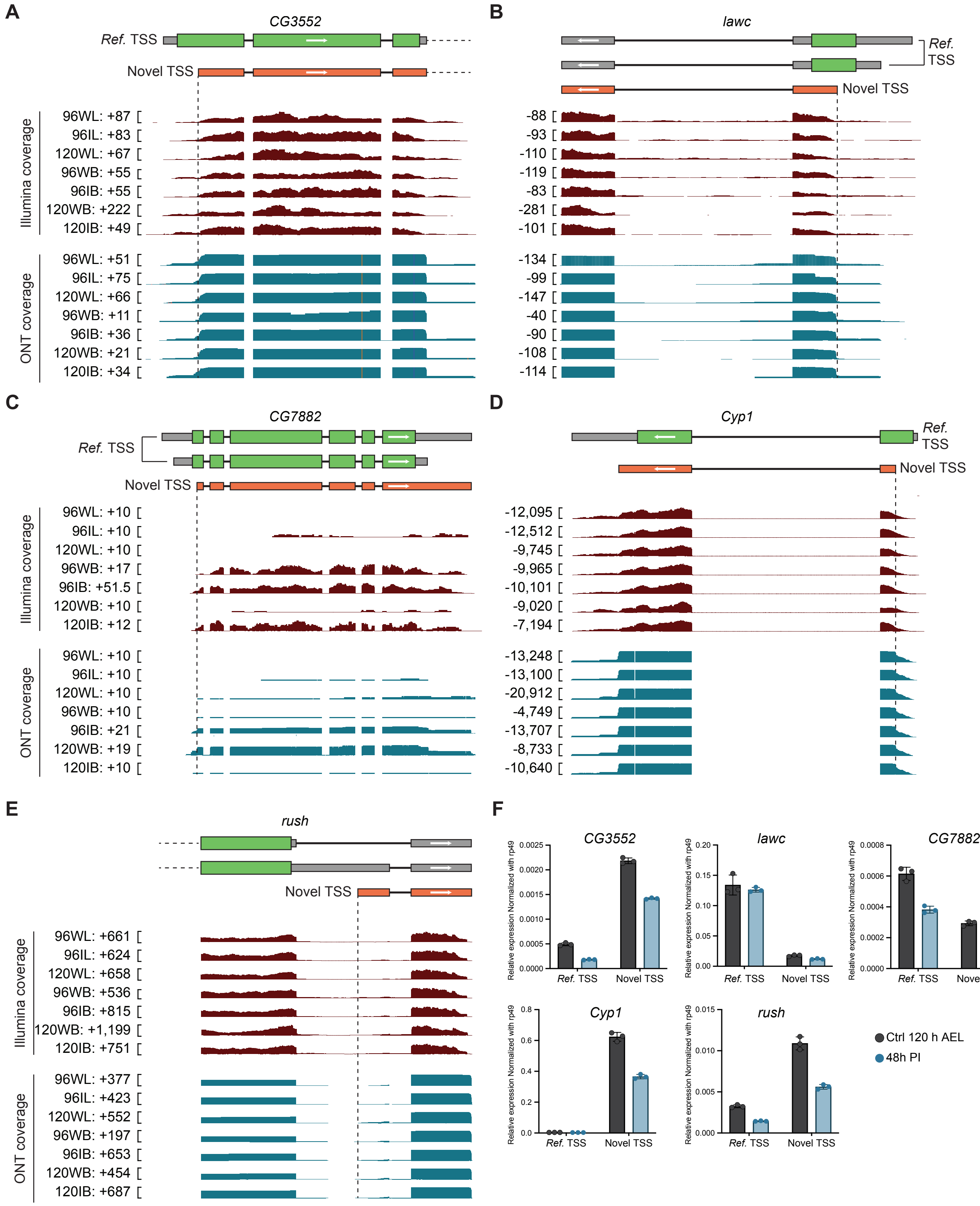

A
CG3552
Ref. TSS
Novel TSS
Illumina coverage
96WL: +87 [
96IL: +83 [
120WL: +67 [
96WB: +55 [
96IB: +55 [
120WB: +222 [
120IB: +49 [
ONT coverage
96WL: +51 [
96IL: +75 [
120WL: +66 [
96WB: +11 [
96IB: +36 [
120WB: +21 [
120IB: +34 [
B
lawc
Ref. TSS
Novel TSS
-88 [
-93 [
-110 [
-119 [
-83 [
-281 [
-101 [
-134 [
-99 [
-147 [
-40 [
-90 [
-108 [
-114 [
C
CG7882
Ref. TSS
Novel TSS
Illumina coverage
96WL: +10 [
96IL: +10 [
120WL: +10 [
96WB: +17 [
96IB: +51.5 [
120WB: +10 [
120IB: +12 [
ONT coverage
96WL: +10 [
96IL: +10 [
120WL: +10 [
96WB: +10 [
96IB: +21 [
120WB: +19 [
120IB: +10 [
D
Cyp1
Ref. TSS
Novel TSS
-12,095 [
-12,512 [
-9,745 [
-9,965 [
-10,101 [
-9,020 [
-7,194 [
-13,248 [
-13,100 [
-20,912 [
-4,749 [
-13,707 [
-8,733 [
-10,640 [
E
rush
Novel TSS
Illumina coverage
96WL: +661 [
96IL: +624 [
120WL: +658 [
96WB: +536 [
96IB: +815 [
120WB: +1,199 [
120IB: +751 [
ONT coverage
96WL: +377 [
96IL: +423 [
120WL: +552 [
96WB: +197 [
96IB: +653 [
120WB: +454 [
120IB: +687 [
F
CG3552
lawc
CG7882
Cyp1
rush
Relative expression Normalized with rp49
Ref. TSS
Novel TSS
Ctrl 120 h AEL
48h PI
