# Supplementary material for: A hybrid transcriptome assembly reveals coding and noncoding landscapes in Drosophila hemocytes
Source: Genetics. 2026 Aug 11;234(2):iyag208. doi: 10.1093/genetics/iyag208 (PMC13643772; doi:10.1093/genetics/iyag208)
Supplement: Supplementary file 1 [file iyag208_supplementary_data.zip › Supplementary_Figure_9_GENETICS-2026-309442.pdf]

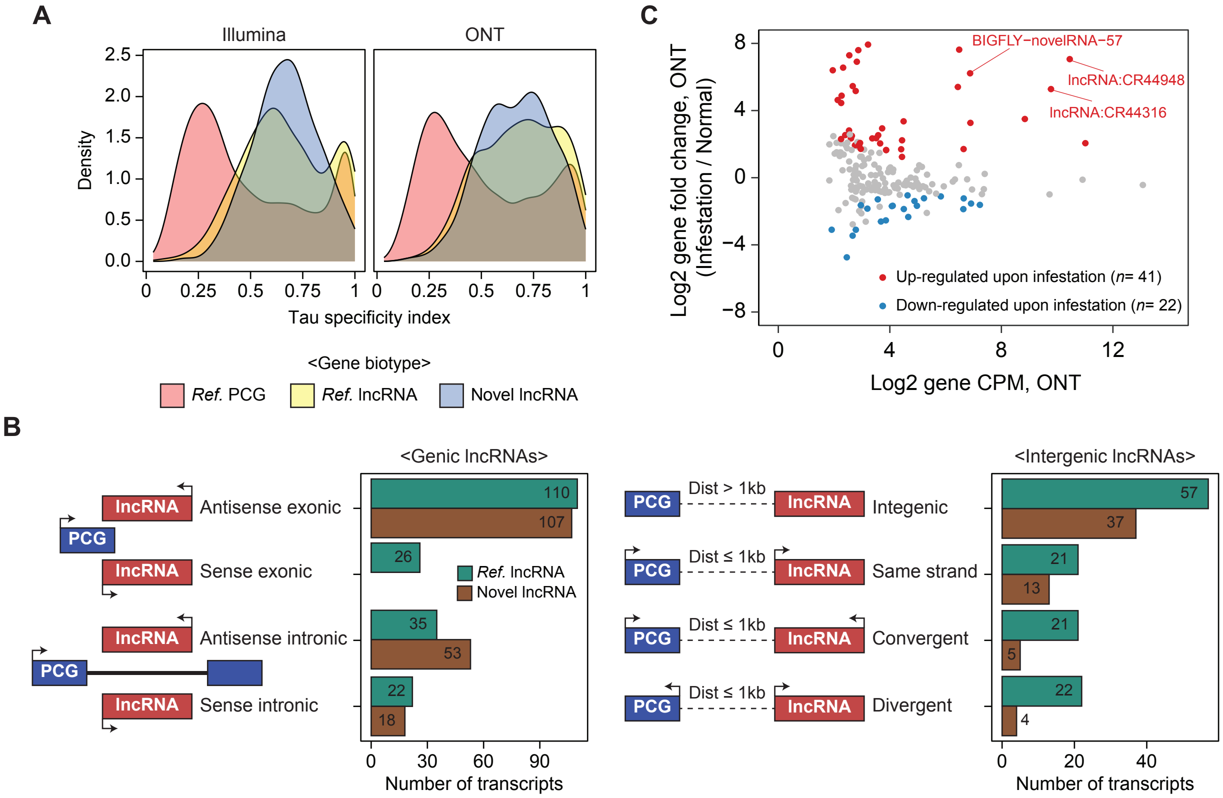

A
Illumina
ONT
Density
Tau specificity index
<Gene biotype>
Ref. PCG
Ref. lncRNA
Novel lncRNA
C
BIGFLY−novelRNA−57
lncRNA:CR44948
lncRNA:CR44316
Log2 gene fold change, ONT (Infestation / Normal)
Up-regulated upon infestation (n= 41)
Down-regulated upon infestation (n= 22)
Log2 gene CPM, ONT
B
<Genic lncRNAs>
lncRNA
PCG
Antisense exonic
Sense exonic
Antisense intronic
Sense intronic
Ref. lncRNA
Novel lncRNA
110
107
26
35
53
22
18
Number of transcripts
<Intergenic lncRNAs>
Dist > 1kb
Dist ≤ 1kb
Integenic
Same strand
Convergent
Divergent
57
37
21
13
21
5
22
4
Number of transcripts
